# Impact of pacifier use and parenting characteristics on toddlers’ vocabulary development

**DOI:** 10.3389/fpsyg.2025.1599801

**Published:** 2025-07-23

**Authors:** Laura Barca, Claudia Mazzuca, Anna M. Borghi

**Affiliations:** ^1^Institute of Cognitive Sciences and Technologies, National Research Council, Rome, Italy; ^2^Department of Dynamic and Clinical Psychology, and Health Studies, Sapienza University of Rome, Rome, Italy

**Keywords:** child’s first vocabulary, pacifier use, parental sense of competence, parental educational style, abstract concepts

## Abstract

**Introduction:**

This study investigated the relationship between pacifier use and early vocabulary development, specifically hypothesizing that pacifier use would correlate with a reduced overall vocabulary size and a specific underrepresentation of abstract words.

**Methods:**

We recruited a sample of 98 typically developing children aged 18-36 months. Data collection included information on pacifier use, feeding habits, parenting styles, parental satisfaction, and vocabulary. Vocabulary was assessed using the MacArthur-Bates Communicative Development Inventory.

**Results:**

Contrary to our initial hypotheses, we found no significant association between pacifier use and either overall vocabulary size or the acquisition of abstract words within this age range. However, we observed a correlation between pacifier use and feeding type, as well as an unexpected positive association with a higher paternal sense of efficacy. Pacifier use did not show a link to specific parenting styles.

**Discussion:**

Our findings suggest that pacifier use in children aged 18-36 months does not negatively impact early vocabulary development. While these results offer valuable insights, further research is warranted to explore the complex factors influencing language development and the potential long-term impact of pacifier use beyond 36 months of age.

## 1 Introduction

Using a pacifier is a very common practice. It is commonly used to calm the child in the first years of life, for example, to facilitate their falling asleep or relieve teething pain. In Italy, the age of pacifier withdrawal typically corresponds with the child attending nursery school, around three years of age. However, in some cases, its use might be prolonged further.

The detrimental effects of non-nutritive sucking habits on oro-facial functions are well known, such as the increased likelihood of alteration in dental arch dimension and malocclusion (Ovsenik, 2009). Some of these defects tend to decrease once the pacifier’s use is abandoned, with an improvement in maxillary and mandibular inter canine widths, breathing, and speech functions 12 months after removal (Scudine et al., 2021). Nevertheless, some defects seem to persist longer. For example, masticatory malfunctions did not self-correct one year after pacifier withdrawal (Scudine et al., 2022), highlighting the need for prevention and habit interruption as early as possible.

Recently, some scholars have wondered whether using a pacifier for a prolonged period (e.g., beyond three years) might hinder the child’s social and linguistic development. The school-age children who participated in these studies no longer used the pacifier, but some of them used it longer than others, affecting some of their emotional competencies (Niedenthal et al., 2012; Rychlowska et al., 2014; Rychlowska and Vanderwert, 2020) and conceptual and linguistic abilities such as describing the meaning of words (Barca et al., 2017) and categorizing them semantically (Barca et al., 2020). Considering these later studies, the detrimental effect of prolonged pacifier use emerged specifically in processing abstract words (and sentences, albeit to a minor extent) compared to concrete and emotional ones. Abstract words (e.g., “thought” and “fantasy”) encompass various words, from social to philosophical to numerical words (Conca et al., 2021). Unlike concrete words (e.g., “chair”), they typically do not have a single object as a referent, and generally, their exemplars are quite diverse (Langland-Hassan et al., 2021). In addition, they evoke less sensorimotor and more inner bodily experiences (interoception) (Connell et al., 2018; Borghi, 2022; Reggin et al., 2021). Critically, abstract words are generally acquired later and through language, and adults feel less confident and more uncertain using them and feel they need/have needed the support of others to acquire them (Mazzuca et al., 2022; Villani et al., 2022). Despite this, they often use them since more than 70% of adult speech consists of abstract words (Lupyan and Winter, 2018).

Because the members of abstract categories are heterogeneous, linguistic labels might help make them more cohesive, and linguistic explanations are crucial for their learning (Borghi et al., 2019). Given language’s crucial role in abstract concept development, we previously investigated whether pacifier use, potentially affecting language acquisition, selectively impacts abstract word processing. Two studies, using definition and categorization tasks, demonstrated that prolonged pacifier use negatively influences abstract concept representation (Barca et al., 2017, 2020).

Our results showed that: (i) abstract words were categorized more slowly (Barca et al., 2020; see also Barca et al., 2024 for similar results on sentence processing), (ii) and their definitions highlight the use of different conceptual relationships compared to concrete and emotional words, indicating more malleable boundaries between different types of concepts (Barca et al., 2017).

The mechanisms by which prolonged pacifier use affects abstract word processing are still under investigation. Linguistic acquisition processes see the complex interplay between motor, sensory, and social dimensions (see Guenther and Vladusich, 2012 for a detailed model of speech development; Hansen, 2017; Pezzulo et al., 2014). The quality of early language experiences appears to be crucial for subsequent language development such that, for example, the amount of speech heard by children is predictive of their language outcomes (Hart and Risley, 1995). In addition to the total amount of linguistic input, other relevant variables influence language development. The pattern of *turn-taking* in proto-linguistic interactions with the mother predicts the breadth of the child’s expressive vocabulary at 24 months (Huber et al., 2023; Nguyen et al., 2023). Ramírez et al. (2023) reported that a high amount of parentese (the acoustically exaggerated, clear, and higher-pitched speech produced by adults when interacting with infants) is associated with lexical diversity and the production of longer sentences when children are 5 years old. Likewise, the first vocalizations produced by children at 6 months predict their expressive speech at 12 months of age (Werwach et al., 2021). Beyond linguistic-specific inputs, even fundamental sensory experiences in early development contribute significantly to the broader cognitive landscape. For instance, recent research on premature newborns highlights how early visual experiences, such as exposure to faces, can shape basic perceptual preferences and influence subsequent cognitive development (de Almeida et al., 2025). This aligns with the understanding that gaze to faces plays a crucial role in supporting face-to-face interaction (Hessels, 2020), which is foundational for social and cognitive learning. In fact, the development of infants’ gaze to faces during early face-to-face contact has been directly linked to their later vocabulary outcomes (Belteki et al., 2022). These findings underscore the critical importance of understanding neurovisual functions for a comprehensive view of early cognitive development (Barca et al., 2010). Such an understanding also extends to how infants process “talking faces” in different contexts, noting that the nature of these visual inputs can vary significantly between screen-based settings and real-life communicative interactions, potentially impacting language learning (Birulés et al., 2023). This highlights the pervasive influence of diverse environmental factors, from basic sensory input to complex social interactions, on the child’s developing brain and, by extension, on language acquisition.

Thus, is it possible that using the pacifier during language acquisition, particularly during social interaction, might have influenced how words were consolidated (Barca, 2021). Using a pacifier during linguistic-social interaction might have several implications: (i) it might interfere with the consolidation of a stable articulatory speech-motor program of words, increasing the variability of the motor representation linked to the pronunciation of the word. Having a words’ speech-motor plan that is not clearly defined might affect their consolidation, making the trajectory of vocabulary increase less steep (i.e., reducing the number of words produced by the child). Such interference might occur at the articulatory level (as holding a pacifier in the mouth limits the co-articulation of speech) and at the auditory level (affecting the child’s self-auditory feedback), thus (ii) it might interfere with the auditory feedback that children get from their own voice, increasing the variability of the phonological representation of the word. The effect of the pacifier might also extend to the *social dimension* of linguistic interaction. Using a pacifier during social interaction might also affect other social actors (Goldstein and West, 1999) who might have more difficulty in understanding the child’s speech (Bloom, 1993), and adapt the child’s direct speech accordingly, for example by using a more simplified language. So (iii) adults might be linguistically less resonant with children using a pacifier, in the same way as they appear to be less emotionally resonant (Rychlowska et al., 2014; Rychlowska and Vanderwert, 2020), and possibly modulate in some way the child-directed speech. They might perceive the child as linguistically less competent and modify the communication accordingly, for example, by reducing the use of abstract language being more anchored to contextual experiences and concepts [see, for example (Pratt et al., 1992)]. Another possible mechanism is based on the “social metacognition” process (Borghi et al., 2019): because abstract words are more complex than concrete ones, children first try to innerly re-explain to themselves the word meaning and, in case they do not find a solution, they might revert to other people to ask them for information and support. Both the inner explanation and the preparation to ask others for information might involve the mouth motor system.

The current studies on pacifier use and linguistic processes present some critical aspects. The measure of pacifier use was operationalized with parental reports gathered several months and, in some cases, years after the child had stopped using the pacifier. Although this measure resulted reliable across studies, some parents might have estimated the frequency of pacifier use less accurately than others; more crucially, their recall of how long their children used the pacifier years ago might not be precise.

Another point worth considering is that children of those studies no longer used the pacifier, so what emerged is a long-term effect of the pacifier (specifically on abstract word processing). An analysis of the child’s linguistic skills performed during the period of pacifier use (i.e., infancy) might reveal the emergence of other phenomena, for example, a concurrent effect of the pacifier in shaping the composition of the child’s first vocabulary. The first words children learn tend to be highly concrete and imageable, where nouns are typically acquired earlier than verbs and adjectives (Hansen, 2017; Rinaldi et al., 2004a; Verhagen et al., 2022). In addition, some characteristics of the child might influence vocabulary acquisition, such as gender, with an advantage of girls over boys (Eriksson et al., 2012; Verhagen et al., 2022), the socioeconomic level of the family, and parental education.

The current study deals with the following questions: (i) Does using a pacifier hinder vocabulary acquisition? and (ii) Does using a pacifier affects to a greater extent the acquisition of abstract words?

To answer these questions, we explored the characteristics of the vocabulary of Italian children aged 18–36 months, as measured by the Italian version of the MacArthur-Bates Communicative Developmental Inventory (Caselli et al., 2007), henceforth referred to as CDI. The CDI is a parental questionnaire for screening early language development, developed with the aim of detecting the expansion of the vocabulary and the changes in its composition up to the emergence of grammar and the construction of the first sentences.

With respect to the first question, we expect children who use the pacifier to have a reduced vocabulary size than children who do not. To date, a handful of studies explored the possible relationship between pacifier use and atypical language development (specifically, developmental speech disorders), and no such relation emerged. That is, articulatory speech disorders (Shotts et al., 2008), phonological disorders (Baker et al., 2018), or speech-sound disorders (Burr et al., 2021) are not associated with pacifier use. However, using a pacifier might still affect the development of another aspect of language, such as vocabulary composition, beyond atypical conditions. For example, children who use the pacifier many hours during the day and in a social context might have a vocabulary with an underrepresentation of abstract words than those who use It less or not at all (the second hypothesis of the study).

**Figure 1:**
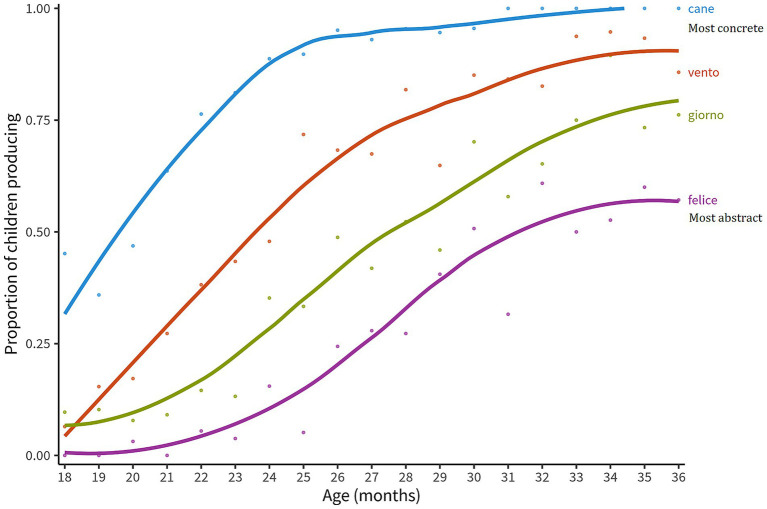
The graph shows the growth curves for four words from the CDI Italian form varied for their level of abstractness/concreteness: “cane” = dog (mean abstractness = 6.9, dev.st = 0.26−most concrete), “vento” = wind (mean abstractness = 4.93, dev.st = 1.75), “giorno” = day (mean abstractness = 3.47, dev.st = 2.2), “felice” = happy (mean abstractness = 3.14, dev.st = 1.75−most abstract). Data taken from the world: http://wordbank.stanford.edu/.

Children’s’ first vocabulary is typically characterized by concrete words, referring to objects, entities, and concepts that are part of the child’s living environment. Abstract words are typically acquired later in life (Bergelson and Swingley, 2013). Indeed, if we consider the trajectories of acquisition of different types of words varied according to the abstractness/concreteness dimension (see Figure 1), a concrete word such as “dog” is present in the expressive vocabulary of most of the children (here 75% of the sample) aged around 22 months. With this same 75% cut-off, words like “wind” consolidate around 29 months of age, whereas for temporal adverbs such as “day” or emotional words such as “happy”, there was greater variability up to 36 months of age.

Thus, the child’s vocabulary gradually enrich with abstract words which are mainly acquired while interacting with others, using language exchanges (e.g., asking them for the meaning of a novel word/concept) and repeating them to ourselves (e.g., via inner speech) (Bellagamba et al., 2022; Borghi et al., 2018; Dove et al., 2022). This process includes the acquisition of socio-moral terms: for example, research shows that by 30 months, toddlers begin to map words like “good” to specific prosocial behaviors, such as helping, though not yet consistently to concepts like fairness in distributive actions (Franchin et al., 2019). When we learn new words in adulthood, if they are abstract we rely mainly on linguistic and social input, whereas for concrete words we rely more on sensorimotor experience (Granito et al., 2015). Additionally, as mentioned above, previous works have shown the impact of a prolonged use of the pacifier to be limited to processing abstract words and not concrete or emotional ones (Barca et al., 2017, 2020). So, further assessing this relation is particularly important, as recent studies suggest that knowing more general words is associated with faster vocabulary learning (Lewis et al., 2021) and better inductive inferences (Suffill et al., 2022).

In order to address whether pacifier use affects word acquisition in general – and abstract words more specifically – we asked parents of children attending nursery schools to fill in the Italian version of the MacArthur Bates CDI (Caselli et al., 2007), aimed at assessing their child’s vocabulary, as well as questionnaires assessing pacifier use and child-family characteristics. Since using a pacifier or not may be related to parental and/or educational styles, we asked parents to fill in some questionnaires assessing parent–child relationship and their social exchange at large.

*Parenting self-esteem*, encompassing the perceived self-efficacy as a parent and the satisfaction derived from parenting, is a pivotal aspect of the parent–child relationship (Johnston and Mash, 1989). Parents who feel confident in their role are more likely to engage in successful parenting strategies (Vance and Brandon, 2017). Parental self-efficacy, for example, is positively related to a child’s healthy behaviors, such as physical activity, a healthy diet with fruit and vegetables (Woods and Nies, 2019), and limited screen time (Kieslinger et al., 2021). In Barca et al. (2024) higher levels of parental satisfaction resulted in fewer months of pacifier use. The months of use of the pacifier also decrease as the sense of efficacy perceived by the parent increases (modest relationship), which reflects the degree to which the parent feels capable and competent in solving problems related to parenting (Johnston and Mash, 1989; Vio et al., 1999). We hypothesize that a similar relationship might be present in the current study.

Another variable that we have considered is the *parental attitude* towards their children. A parental attitude such as the encouragement of initiative-taking is positively associated with sociability-assertiveness and negatively associated with academic problems in school-age children (Chen et al., 2000). This aligns with a broader understanding that parental approaches significantly influence various aspects of child development. For instance, recent work by Geraci et al. (2023) highlights how early parental education can shape even fundamental socio-cognitive skills, such as infants’ sense of fairness and justice, underscoring the deep and pervasive impact of parental influence from the very beginning of life. The quality of socio-cognitive engagement between parents and their children is directly linked to positive developmental outcomes. When parents and children engage in joint activities like storytelling and interactive play, it demonstrably improves children’s language and cognitive skills (Schulze and Saalbach, 2022; Sohr-Preston et al., 2013). This perspective is further supported by Rivero et al. (2023), which highlights the significant relations between positive parenting behavior during play and child language development at early ages. Their findings reinforce the crucial role of engaged and qualitative parent–child interactions in fostering early language acquisition and cognitive growth.

Here, we used the Italian version (Venuti and Senese, 2007) of the questionnaire developed by Bornstein et al. (2001), which measures how parents rate their attitudes toward their children distinguishing between social interaction, didactic interaction, and interaction centered on the discipline. We hypothesize that pacifier use might be associated with a parental attitude more focused on discipline and rule learning.

## 2 Materials and methods

The data were collected at three nurseries in central Italy [two nurseries were located in a suburb of Rome, one in Venafro (province of Isernia)]. A meeting was organized with the parents to present the research. On this occasion, the experimental materials were delivered to the parents who provided informed consent to participate in the study. Materials comprised the following questionnaires: an enrolling questionnaire, the MacArthur-Bates CDI, the Parenting sense of competence questionnaire, and the Parenting style questionnaire (see the next section for details). The experimental procedure has been approved by the relevant Ethical Committee (no. 0001986, 03/06/2015). Statistical analyses were performed using R (R Core Team, 2021).

### 2.1 Enrolling questionnaire

The questionnaire was aimed to gather information on the children’s feeding type (e.g., exclusive breastfeeding or if he/she was weaned) and pacifier use (e.g., if the child used it and how often).

### 2.2 MacArthur-bates communicative development inventory (CDI)

This is a parental questionnaire for screening early language development of children aged 18–36 months. The form is divided into three parts. The first part, “Gestures and words”, includes a list of 670 words belonging to 23 different categories, as content words (i.e., Sounds and voices of nature, Animals, Vehicles, Food and drinks, Clothing, Body parts, Objects of use family, Furniture and rooms and objects of the house, Outdoors, Places to go, People, Routines, Verbs, Adjectives, and qualities, Adverbs-Time expressions), Pronouns, Interrogatives, Prepositions, Articles and quantifiers, Auxiliary and Modal Verbs, Conjunctions, Adverbs—Place and quantity expressions. Six questions follow, aimed at evaluating the capacity for spatial and temporal contextualization. The second part, “How children use grammar”, investigates the ability to use the singular/plural of nouns, the gender, the number of adjectives, and the singular/plural conjugation of verbs. The third part, “How children use sentences”, evaluates the complexity and length of the sentences produced. For example, the syntactic complexity of the sentences is evaluated in the passage from nuclear sentences to those with a more complex structure. Here we discuss the data relating to the first part of the inventory (i.e., the list of 670 words).

### 2.3 Parenting sense of competence questionnaire

We used the Italian version (Vio et al., 1999) of the questionnaire developed by Johnston and Mash (1989). It is composed of 16 questions: 9 items concerning the parental sense of *Satisfaction* (an affective dimension reflecting the degree of parental frustration, anxiety, and motivation in the parenting role) and 7 items concerning the parental sense of *Efficacy* (an instrumental dimension reflecting the parental sense of competence, capability of problem-solving in the parenting role).

### 2.4 Parenting style questionnaire

We used the Italian version (Venuti and Senese, 2007) of the questionnaire developed by Bornstein et al. (2001), which measures how parents rate their attitudes toward their children. The behaviors evaluated concern three different types of parent–child interactions. *Social interaction* concerns the interpersonal exchanges in the parent–child dyad and involves the expression of sensitivity, reciprocity, and affection. Overall, the social style fosters the development of children’s social competencies (Chen et al., 2000). *Didactic interaction* reflects how parents stimulate the children’s awareness of objects and events in the environment external to the dyad, offering them the opportunity to observe, imitate and learn. The didactic type of interaction promotes the child’s cognitive and linguistic development (Tamis-LeMonda and Bornstein, 1989). Finally, the *interaction centered on Discipline* concerns how much the parent encourages the development of attitudes complying with conventions, rules, and respect for authority.

The CDI inventory and the other questionnaires were delivered to the parents at the School meeting, in paper format and organized in a single dossier. We asked for the completion of either the father or the mother (not both), using the appropriate forms when necessary (as in the case of the parental questionnaires), with the request to return it to the School within the following two weeks.

## 3 Results

### 3.1 Characteristics of the children’s sample

The total sample comprises the data of 105 children. From this, the data of 6 children aged 17 months (younger than the CDI normative sample), were not considered, as those of a 46-month-old child (older than the normative sample). The final sample, thus, comprises the data of 98 children (45% girls, 55% boys).

### 3.2 Demographic characteristics of the sample

The group of children considered has a typical development, and parents did not report any diagnosis of atypical development, sensory, cognitive, or language deficits. Most of them were born full term (65%), in 17% of cases they were born preterm (without associated deficit), and in 15% of cases the information was not provided.

Most of the parents indicated Italian as their nationality (75%), and 7% indicated other nationalities (i.e., Romanian, Eritrean, Nigerian, Ukraine). Fifteen percent of the sample did not report this information. Accordingly, the majority of children were only exposed to Italian, and 16% also to other languages (i.e., English, Spanish, Eritrean, Romanian, Russian, Ukrainian).

### 3.3 Frequency of pacifier use and other non-nutritive sucking behavior

Overall, the distribution of pacifier use is very similar between females and males (see the Appendix for details). The pacifier is used by most of the children in the group (68%), and the majority are currently using it (47%, age range = 21–31 months). Some children (21%) have used it for some time (from 1 to 12 months) but no longer use it.

In this sample, the pacifier is used often both during the day and at night (43%), and 4% less frequently. Although we have not directly investigated the contexts and situations of pacifier use, it is reasonable to hypothesize that these children also used the pacifier in social contexts and interacting with others. In contrast, only 5% of the children use it only at night.

As for other non-nutritive sucking behaviors, only 4 out of 99 children indulge in thumb sucking (three of them also use a pacifier). In addition, 11 other children use other objects for non-nutritive sucking, such as a bottle teat, pieces of fabric, or some hard plastic toy (which might be an attempt to relieve the discomfort of teething).

### 3.4 Infant feeding habit

The majority of children were fed through exclusive breastfeeding (51%). The percentages of children fed with formula milk or with a mixed type of feeding were very similar (26 and 23%, respectively). It is important to note that we did not collect specific data on the duration of any feeding type (exclusive breastfeeding, formula feeding, or mixed feeding). While exclusive breastfeeding likely continued until the introduction of solid foods, the exact duration was not recorded for this group or for those with mixed feeding patterns.

Most of the children who use the pacifier have been breastfed (43%) or had a mixed feeding type (31%) followed by 26% who were fed with formula milk. All the children were weaned at the time of the study (i.e., switched to solid feeding), except one 24-month-old child.

To test for a possible association between infant feeding type and pacifier use, we performed a Bayesian test of association (the expected frequencies were too small due to missing cases, and the resulting Chi-squared approximation might lack reliability). We used the R’s BayesFactor package (Morey et al., 2022) with default priors and a Poisson sampling plan (i.e., when rows, columns, or sample size are not fixed *a priori*). The resulting Bayes factor (BF) for feeding type is 18.56 (the odds for the alternative hypothesis against the null are about 19:1), indicating that exclusive breastfeeding is associated with a higher likelihood of pacifier use in this sample. In other words, there is strong evidence of a relationship between pacifier use and feeding type.

### 3.5 Socio-cultural level of the family

The socio-cultural level of the family was determined based on the highest educational qualification achieved by one of the parents. We considered three levels of education: primary school diploma, high school diploma, and bachelor’s degree. Overall, the socio-cultural level of the family of the sample is quite high since the majority of them have at least one parent with a higher educational level (i.e., 53% with a high school diploma and 40% with a bachelor’s degree). Seven percent of parents graduated from primary school.

Since maternal education has been suggested as a critical factor for pacifier use, with a higher level of maternal education leading to a younger age of pacifier withdrawal (Korlahalli et al., 2014), Table 1 reports the percentages of pacifier use according to maternal and paternal educational level.

**Table 1:** Percentages of pacifier use and parental level of education.

	Mother			Father		
Pacifier	Bachelor degree	Primary school	High school	Bachelor degree	Primary school	High school
No	24	33	32	30	25	33
Yes	76	67	62	70	75	67

The high prevalence of pacifier use suggests a shared parenting practice across education levels for both mothers and fathers in this sample. While a Bachelor’s degree is the most common educational attainment for both parents, mothers exhibit a slightly more balanced distribution across the three educational categories compared to fathers, where the Bachelor’s degree represents a larger proportion.

Separate Bayesian tests of association were conducted for the educational level of the mother and father. The resulting Bayes factor (BF) for maternal education is 0.25 to 1 in favor of the alternative hypothesis, which indicates that there is no evidence for the non-independence of pacifier use and maternal education. In other words, pacifier use is not associated with different levels of maternal education. Likewise, no evidence emerged of the association also with the paternal level of education (BF = 0.17).

### 3.6 Parenting questionnaires

Most of the parenting questionnaires were filled in by the mothers (52%), 29% by both parents, and 12% only by the fathers (with 7% of missing data). Due to the study design allowing either one or both parents to participate, paired data for both mothers and fathers was available in only 29% of cases, precluding a reliable analysis of within-family associations in their sense of satisfaction or efficacy; this represents a potential avenue for future research with a design focused on dyadic parental responses.

#### 3.6.1 Pacifier use and parenting sense of competence (satisfaction and efficacy)

The questionnaire evaluates the parents’ sense of satisfaction and effectiveness in their parenting role. Considering the responses of the mothers, 19 out of 98 responses were missing (NA), which represents 19.39% of the data. For the available data, most respondents have a high level of sense of *Satisfaction* in parenting (i.e., 19% excellent, 30% good, 21% sufficient), with a reduced group experiencing inadequate (11%) and problematic (19%) levels.

Regarding the subscale of Efficacy, we observed considerable missing data, with 24 out of 98 potential responses (24.5% of the total sample) being incomplete or entirely missing (NA). We acknowledge that this proportion can affect the strength and generalizability of our findings, especially for analyses that rely on smaller effective subsamples.

Despite these data limitations, descriptive analysis of the available data for mothers revealed that most reported a high sense of efficacy: 30% described it as “excellent,” 36% as “good,” and 19% as “sufficient.” A smaller proportion indicated a inefficient sense of effectiveness (6% “inadequate” and 9% “problematic”). Consistent with these observations, Bayesian analysis indicated no association between pacifier use and different levels of maternal sense of satisfaction or efficacy (BF = 0.94 and 0.32, respectively).

Regarding the paternal questionnaire, we note a substantial amount of missing data, with 64 out of 98 potential responses being incomplete or entirely missing (NA). Among the fathers who completed the form, most reported high satisfaction with their parenting role (26% excellent, 26% good, 22% sufficient level), while a smaller percentage indicated an inadequate (17%) or problematic (9%) sense of Satisfaction. For the fathers’ sense of Efficacy, we again observed a substantial amount of missing data, with 66 out of 98 potential responses being incomplete or entirely missing (NA). Among the fathers who provided data, the majority reported a positive sense of Efficacy (50% excellent, 14% good, 27% sufficient), with only a small minority indicating an inadequate (5%) or problematic (5%) sense of Efficacy. Table 2 reports the percentages of pacifier use according to maternal and paternal levels of satisfaction and sense of efficacy.

**Table 2:** Percentages of pacifier use and parental sense of satisfaction and efficacy.

Sense of satisfaction										
	Mother					Father				
Pacifer	Inad	Prob	Suff	Good	Opti	Inad	Prob	Suff	Good	Opti
No	14	21	45	23	21	43	0	17	45	25
Yes	86	79	55	77	79	57	100	83	55	75

Sense of efficacy										
	Mother					Father				
Pacifier	Inad	Prob	Suff	Good	Opti	Inad	Prob	Suff	Good	Opti
No	25	29	29	37	16	50	50	14	67	8
Yes	75	71	71	63	84	50	50	86	33	92

Inad = Inadequate; Prob = problematic; Suff = Sufficient; Good = good; Opti = Optimal.

As for maternal scores, the size of the Bayes factor indicates that there is no evidence of the non-independence between pacifier use and maternal sense of satisfaction (BF = 0.94), and sense of efficacy (BF = 0.32). As for paternal scores, the test of association produced a Bayes factor of 1:1 in favor of a relationship between pacifier use and paternal sense of satisfaction (no evidence), and evidence suggesting a strong relationship (BF = 33:1) for the non-independence of pacifier use and paternal sense of efficacy, though interpretation of “robustness” is tempered by the sample size/missing data.

While the data show no apparent connection between pacifier use and either mothers’ satisfaction/efficacy or fathers’ satisfaction, it does point to a possible link between pacifier use and fathers’ sense of efficacy. However, the small subsample for these analyses warrants caution in interpreting the strength of the evidence, particularly for paternal sense of efficacy.

#### 3.6.2 Pacifier use and parental interactive style (social, didactic, and disciplinary)

The items of the questionnaires tackle three areas of parent–child interaction: *social area*, *didactic area*, and *disciplinary area*. Overall, the mothers in this sample report stimulating their children mostly in the social and disciplinary domains (52 and 43%, respectively), and to a minor extent in the didactic domain (5%). We note that 14 out of 98 potential responses for this questionnaire were incomplete or entirely missing (NA).

For fathers, we observed a substantial amount of missing data for this questionnaire, with 62 out of 98 potential responses being incomplete or entirely missing (NA). Among those who provided data, fathers primarily stimulated the disciplinary domain (53%), followed by the social (31%) and didactic (17%) domains. Table 3 presents percentages of pacifier use according to maternal and paternal parenting styles.

**Table 3:** Percentages of pacifier use and parenting style.

	Mother			Father		
Pacifier	Didactic style	Disciplinary style	Social style	Didactic style	Disciplinary style	Social style
No	25	36	25	33	42	50
Yes	75	64	75	67	58	50

Looking at the row percentages, we can observe the distribution of parenting styles within the ‘No Pacifier Use’ and ‘Yes Pacifier Use’ groups for both mothers and fathers. For mothers who reported no pacifier use, there was a relatively even split across the Didactic (25%), Disciplinary (36%), and Social (25%) styles. However, when mothers reported pacifier use by their children, there was a notable shift towards higher percentages in the Didactic (75%) and Social (75%) styles, with the Disciplinary style also being prevalent (64%) over the “No Pacifier” group.

Examining the fathers’ row percentages reveals a different pattern. Among fathers who reported no pacifier use, the Social style shows the highest percentage (50%), followed by the Disciplinary (42%) and then the Didactic style (33%). For fathers who reported pacifier use, the percentages across the styles are somewhat more balanced, with Didactic (67%), Disciplinary (58%), and Social (50%) all representing a substantial portion.

Considering the column percentages, we see the proportion of mothers and fathers within each parenting style based on pacifier use. For the Didactic style, a larger percentage of those who employ this style are mothers, regardless of pacifier use (no: 27.0%, yes: 52.6%). The Disciplinary style also shows a higher representation of mothers in both pacifier use groups (no: 30.5%, yes: 52.9%). Conversely, the Social style presents a more balanced distribution between mothers and fathers in the ‘No Pacifier’ group (mothers: 22.7%, fathers: 45.5%), while in the ‘Yes Pacifier’ group, mothers still constitute a slightly larger share (mothers: 42.9%, fathers: 42.9%).

In summary, the data suggests potential associations between pacifier use and reported parenting styles, with mothers who use the pacifiers with their children tending to report higher use of Didactic and Social styles. Fathers, on the other hand, show a more pronounced Social style in the absence of pacifier use. However, a test of association produced a Bayes factor of 0.3 to 1 in favor of a relationship between pacifier use and maternal parenting style, and 0.5:1 with a paternal parenting style (i.e., no evidence in favor of the association).

To summarize, within this study group, the use of the pacifier does not appear to be associated with the infant’s feeding habits, the parents’ level of education, or their parenting style of interaction. Similarly, no evidence emerged regarding a possible association between pacifier use and a maternal sense of satisfaction and efficacy.

However, an association did emerge between pacifier use and a paternal sense of efficacy. It is important to note that, while this association is statistically significant, the interpretation of its robustness is tempered by the considerable amount of missing data within the paternal questionnaires. This limitation means the findings should be considered with appropriate caution.

### 3.7 The child’s first vocabulary and pacifier use

#### 3.7.1 Pacifier use and CDI lexical indexes

In this section we explore whether pacifier use affects some features of the child’s first vocabulary as measured by the McArthur CDI questionnaire (Caselli et al., 2007).

In general, this sample of children confirms a high variability in language skills, in line with the normative data of this development stage (Caselli et al., 2007). While the sample size varies considerably across age groups (e.g., only 4 children are represented at 18 months), some general patterns emerge (data are reported in the Appendix; Table 2). For example, 31.63% of the sample has a number of words greater than or equal to the 50th centiles of the normative sample. At 18 months, the few children observed are primarily positioned in the lower percentile groups (<C5, C5, C10). As chronological age increases, there tends to be a broader distribution of children across more percentile bins, suggesting a wider range of language development within older age groups. For example, at 30 months, children are found across multiple categories, including C5, C10, C25, C50, C75, C90, and C95. It’s also noteworthy that the age groups 23 months (9 children) and 30 months (11 children) represent the largest subgroups within this dataset, providing more robust observations for these specific age ranges. Overall, approximately 37.76% of the children in your sample have a vocabulary score below the 10th percentile according to the Italian CDI normative sample. This indicates a significant portion of the children fall into the lower end of the vocabulary production range A vocabulary size lower than the tenth centile (based on sex and gender) is typically considered an indication of a delay in expressive language (Verhagen et al., 2022). However, since a diagnosis of language deficit needs to be certified with a thorough evaluation of the child’s linguistic abilities [see also (Eriksson, 2023)], we refrain from considering these results as an indication of an atypical linguistic trajectory.

From the scores of the vocabulary lists, it is possible to derive an *age of lexical development* or lexical age (comparing the number of words produced by the child with the values relating to the 50th percentile) and a *lexical quotient* (dividing the lexical age by the chronological age and multiplying by 100), allowing evaluating if the child’s production is in line with the reference group. In our sample, the Age of Lexical Development is correlated with the chronological age (Pearson r = 0.65, *p* < 0.001), and some variability in the alignment of these measures is observed, as expected for this age group. Specifically, only 28% of the children have an Age of Lexical Development equal to or greater than their chronological age. The majority of the children of the sample (68% of cases) have a lexical age lower than expected for their age, and in some cases (*n* = 19) with a gap of up to 18 months (the lower edge of the CDI normative sample). Taking into account the Lexical Quotient, most children (60%) have an adequate score (equal to/greater than 80).

Thus, this group of children has an expressive vocabulary in line with normative data.

Taking into account the months of pacifier use, this is not correlated with Age of Lexical Development (Pearson r = 0.02, ns), nor with the Lexical Quotients (Pearson r = −0.10, ns).

#### 3.7.2 Pacifier use and vocabulary breadth

To assess whether pacifier use hinders children’s linguistic production beyond the CDI indexes, we modeled the number of words produced by the child as a function of months of pacifier use. We implemented a Bayesian Poisson regression using “brms” R’s package (Bürkner, 2017), with months of pacifier use as a continuous predictor, and months of chronological age as an offset variable, to account for the variability of the age of our sample. A visual inspection of the model showed that the model did not align well with observed data, due to overdispersion. So, we fitted a second model with the same structure using a negative binomial regression, and compared the two models with leave-one-out cross-validation (Vehtari et al., 2017). The difference in the predictive accuracy of the two models showed the first model was consistently worse (ELPD = −9195.2). Thus, in what follows we discuss results from the second model. We found no evidence for an effect of pacifier use on the total number of words produced by children, b = 0, 95% CI [−0.02, 0.02]. To quantify the evidence in favor of the null hypothesis (e.g., the months of exposure to a pacifier do not have any impact on linguistic production) we computed Evidence Ratio, which showed the “no difference” hypothesis was 29.27 times more plausible than the alternative hypothesis, with a posterior probability of 97% (which can confidently be interpreted as good evidence favoring the “no difference” hypothesis).

To test whether there was a difference in the linguistic competence of children depending on gender, we also fitted the same model adding gender as a further predictor. Data from one child were removed for the purposes of this analysis, as parents did not report their gender. The final sample is therefore composed of 96 children (43 females, *M*_age_ = 26.76 months, *SD* = 5.24, 53 males, *M*_age_ = 27.22 months, *SD* = 5.52). We did not find any difference between females and males in terms of the overall number of words produced, b males = 0.07, 95% CI [−0.31, 0.44].

#### 3.7.3 Pacifier use and vocabulary composition

To explore possible differences in vocabulary composition based on pacifier use, we only considered children aged more than 24 months, being the most prolific linguistic period of infancy. Thus, we divided the total sample of children older than 24 months (*N* = 64) into two groups based on the median of the “percentage of life spent using a pacifier” variable. Specifically, after calculating this percentage for each participant, we found the median to be 76%. Based on this, we have formed two distinct groups: Group 1 (lower pacifier use), including all children with a percentage of pacifier use less than 76% (*n* = 32), and Group 2 (higher pacifier use), including all children with a percentage of pacifier use equal to or greater than 76% (*n* = 32). Children who used the pacifier less (group 1) produced 11,057 total occurrences; children who used the pacifier for a longer period (group 2) produced 10,339 occurrences.

To investigate whether there was a significant difference in the total number of unique words produced between children in Group 1 (Lower Pacifier Use) and Group 2 (Higher Pacifier Use), we performed a Negative Binomial regression analysis in R 4.2.0. This model is appropriate for count data and explicitly accounts for overdispersion, which was identified in a preliminary Poisson model. We included the child’s age (in months) as a covariate in the model to control for its potential influence on vocabulary breadth. The model indicates significant differences in the rate of unique words produced by age, but not by group. Group 1 (lower pacifier use) served as the reference group. The coefficient for Group 2 was 0.22054 (standard error = 0.22834, z-value = 0.966, *p*-value = 0.33413). This translates to an Incidence Rate Ratio (IRR) of 1.246748. This IRR suggests that the rate of unique words produced in Group 2 (higher pacifier use) is approximately 1.25 times (or 25% higher) than the rate observed in Group 1, controlling for age. The 95% Confidence Interval (CI) for the IRR was [0.7725497, 2.010318]. Crucially, this interval includes 1, indicating that the observed difference is not statistically significant. The coefficient for age was 0.09589 (Standard Error = 0.02955, z-value = 3.245, *p*-value = 0.00117). This translates to an Incidence Rate Ratio (IRR) of 1.100634. This IRR indicates that for every one-unit increase in age (one month), the rate of unique words produced increases by approximately 10.06%, controlling for group. The 95% CI for the IRR was [1.0332916, 1.172080] confirming the statistical significance of age. To summarize, children in Group 2 (higher pacifier use) did not produce a statistically significantly different rate of unique words compared to children in Group 1 (lower pacifier use), once age was accounted for. However, as expected, the rate of unique words produced significantly increases with age, regardless of pacifier use.

Next, we utilized Negative Binomial regression models (glm.nb) to investigate the relationship between pacifier use (Group 1: lower pacifier use; Group 2: higher pacifier use) and the number of unique words produced by children across various semantic categories, while controlling for age (see the Appendix for detailed output). Age consistently emerged as a highly significant positive predictor for the acquisition of unique words in almost all examined lexical categories. This finding aligns with developmental expectations, confirming that children’s vocabulary generally expands with increasing age. The positive coefficients indicate that, for each unit increase in age (likely months, given the context of child vocabulary development), the expected count of unique words in these categories increases exponentially. For the vast majority of lexical categories, there was no statistically significant difference in the number of unique words produced between children who used a pacifier for longer (Group 2) and those who did use it less (Group 1). This suggests that pacifier use, as defined by the ‘percentage of usage’ in this analysis, does not appear to have a widespread negative (or positive) impact on unique word acquisition across these categories. The only category where pacifier use showed a statistically significant effect was *Routines*. In this category, children in Group 2 (pacifier users) demonstrated a significantly higher number of unique words related to routines compared to Group 1. This is an interesting and specific finding that warrants further exploration to understand the underlying mechanisms or contextual factors that might explain this particular association. A few categories, such as *Auxiliary* (for Group 2), *Familiar Objects* and *Verbs* (for age), showed marginally significant *p*-values (e.g., p ≈ 0.05–0.06). While not reaching the conventional threshold for statistical significance, these suggest a potential trend that could be further investigated with larger sample sizes or different analytical approaches. For the *Articles* and *Conjunctions* categories, neither age nor pacifier use demonstrated a significant impact on the number of unique words. This indicates that the acquisition of these specific grammatical categories might follow different developmental trajectories or be less influenced by the demographic and behavioral factors examined in this study.

The findings predominantly indicate that age is the strongest determinant of vocabulary growth across most lexical categories in this sample. While pacifier use does not generally appear to hinder unique word production, its specific association with a higher number of unique words in the routines category presents an intriguing area for future research. The successful application of Negative Binomial models effectively addressed the initial overdispersion, providing robust and reliable estimates for the observed relationships.

#### 3.7.4 Pacifier use and abstract words

**Figure 2:**
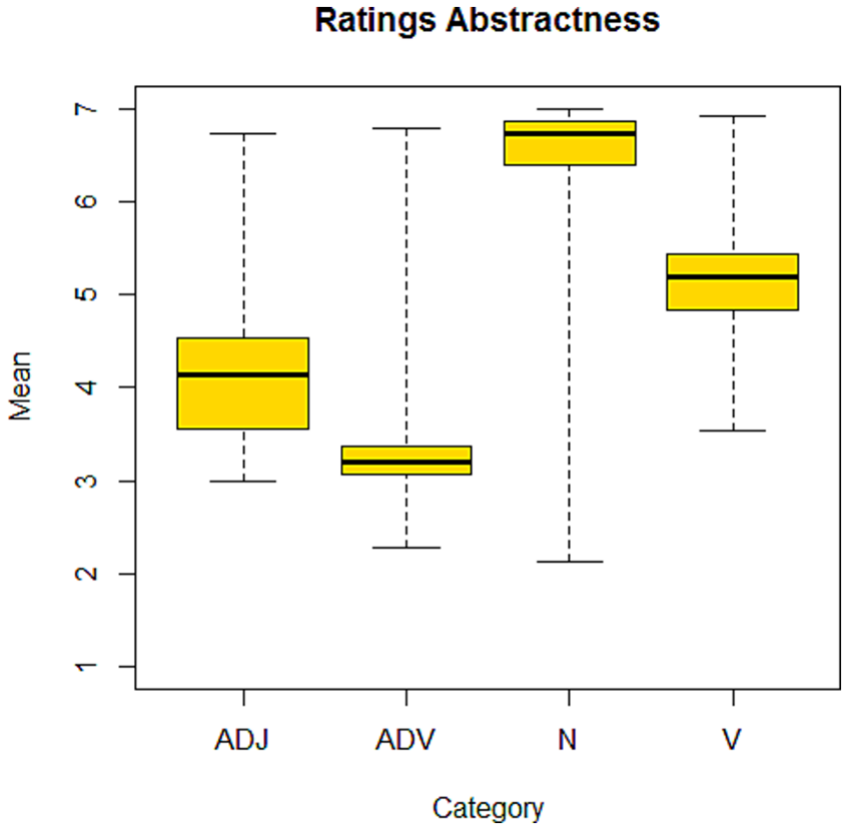
Mean abstractness ratings (low values—highly abstract, high values—poorly abstract) for adjectives (ADJ), adverbs (ADV), nouns (N), and verbs (V) of the CDI.

One objective of the study is to assess whether pacifier uses might hinder the acquisition of abstract words. However, overall, the child’s first vocabulary is poor in abstract words. So, to study the abstractness/concreteness dimension we collected abstractness values (high/low) for the items of the CDI. Italian university students (*N* = 15, *M*_age_ = 32.71; *SD* = 10.16; all females)^1^ were asked to rate the items from the vocabulary list of the CDI on a Likert scale ranging from 1 (highly abstract) to 7 (poorly abstract). Abstractness ratings were not collected for the entire CDI vocabulary. Specifically, 637 out of 675 stimuli were rated, with the omitted items comprising 2 verbs, 7 articles, 17 auxiliaries, and 5 conjunctions. Data are reported in the Appendix (Table 3). Figure 2 reports the average values of abstractness for the macro categories of adverbs, adjectives, nouns, and verbs contained in the child’s first vocabulary.

Figure 2 shows that although this is a set of very simple words, university students have modulated their abstractness ratings assessing the adverbs and adjectives of the CDI as the more abstract categories, while verbs and nouns were judged to be less abstract.

Overall, the CDI set of stimuli is predominantly composed of categories with reduced levels of abstractness, such as verbs (15.91%), food and drinks (10.55%), and familiar objects (7.72%). An exception is constituted by adjectives and qualities, which received an average rating of abstractness of 3.99 (depicted in Figure 2) and account for the 9.92% of the overall set of stimuli.

To investigate whether Group 1’s vocabulary was more abstract than Group 2’s, we first calculated the mean abstractness (ABS) exclusively for words actually produced by each child. This approach yielded a “mean abstractness value” specific to each child’s active vocabulary, which varied across individuals and allowed for direct group comparisons. Subsequently, to determine if there was a statistically significant difference in this mean vocabulary abstractness between children in Group 1 (lower pacifier use) and Group 2 (higher pacifier use), we conducted a Linear Regression analysis in R 4.0.2 (chosen because the dependent variable, the mean abstractness rating of produced words, is a continuous variable). We included the child’s age (in months) as a covariate in the model to control for its potential influence on vocabulary abstractness. Group 1 (Lower Pacifier Use) served as the reference group in this analysis. The estimated coefficient for Group 2 (Higher Pacifier Use) was 0.002667 (SE = 0.077221, t-value = 0.035, *p*-value = 0.973; 95% CI [−0.1518, 0.1571]). Given the high p-value (well above 0.05) and the fact that the confidence interval includes zero, we conclude that there is no statistically significant difference in the mean abstractness of produced vocabulary between children in Group 2 and Group 1, after controlling for age. The observed difference is negligible and likely attributable to random chance. Considering the Age Effect, the estimated coefficient for age was 0.011778 (SE = 0.009991, t-value = 1.179, *p*-value = 0.243; 95% CI [−0.0082, 0.0318]). Consistent with the group effect, the p-value for age is above 0.05 and the confidence interval includes zero, indicating that age does not have a statistically significant effect on the mean abstractness of produced vocabulary in our sample. This suggests that, within our sample, the mean abstractness of produced vocabulary does not significantly change with increasing age. Notably, the overall fit of the model was very low (Multiple R-squared = 0.02295, Adjusted R-squared = −0.009083; F-statistic = 0.7165 (with 2 and 61 degrees of freedom), *p*-value = 0.4925). These values collectively indicate that the model as a whole is not statistically significant and explains a negligible portion of the variance in the mean abstractness of produced vocabulary.

## 4 General discussion

In this study, we investigated whether using a pacifier during language acquisition might affect the characteristics of the child’s first vocabulary. Previous studies have shown that the age of pacifier withdrawal affects linguistic processes later in life so that school-age children who used the pacifier for a longer period were slower in the processing of abstract words (Barca et al., 2017, 2020) and sentences (Barca et al., 2024) compared to children who used it less or did not use it all. Based on this evidence, we hypothesized a detrimental effect of pacifier use on the child’s first vocabulary, such as a reduced vocabulary size in children who use the pacifier longer. We also expected a modulation of vocabulary composition, with an underrepresentation of abstract words (e.g., temporal adverbs) in children who use the pacifier compared to those who do not. In contrast, other categories—such as body parts—may not be affected. Finally, we also aimed to identify a possible relationship between parental characteristics (e.g., a sense of satisfaction and effectiveness in parenting) and particular styles of parent–child interaction and pacifier use. We start by discussing these latter data to get an overview of pacifier use in this sample of children and their families.

Overall, the percentage of pacifier use is quite high and in line with previous studies on Italian samples (Barca et al., 2017, 2020, 2024). The family habits that we considered in this study, such as how the infant was fed in infancy, do not appear to be related to pacifier use. Most of the children were exclusively breastfed, with the rest of the group with a similar percentage with mixed feeding type or formula milk. Pacifier use appears to be related to these feeding types, with greater use among children who were breastfed or had mixed feeding.

Some socio-demographic characteristics of the family, such as a low level of maternal education, have previously been related to pacifier use so the mean age of pacifier withdrawal decreased as the level of maternal education increased (Korlahalli et al., 2014). Overall, the educational level of the parental sample is quite high, and we do not confirm an association between pacifier use and maternal education. However, the studies we were referring to were conducted with older children (4 to 8 years in Korlahalli et al., 2014), where the greater variability in the age of pacifier withdrawal might allow the detection of differences that remain subtle in our group. In addition, here we cannot consider the current use of the pacifier as a ‘prolonged use’ as those who use it are still in a typical range, being at most 36 months of age. Pacifier use is not associated either with the paternal level of education.

Parents who feel confident in their parenting role, and who feel they are responding effectively to their child’s needs and behaviors are also more likely to engage in parenting successful strategies and healthy behavior (Johnston and Mash, 1989; Kieslinger et al., 2021; Vance and Brandon, 2017; Woods and Nies, 2019). Preliminary evidence suggests that—in school-age children—the age of pacifier withdrawal decreases with an increased sense of parental efficacy (Barca et al., 2024). We hypothesized that a similar relationship might be confirmed with younger children, that is, high levels of parental sense of satisfaction and efficacy might result in not using the pacifier. Here, we found evidence for such an association but in an unexpected direction. Overall, parental sense of satisfaction and efficacy were quite high in this group of parents. And we found that pacifier use was associated only with the paternal sense of efficacy, so its’ use in young children appears to be associated with high levels of paternal sense of efficacy. The observed relation between pacifier use and paternal sense of efficacy should be considered preliminary due to the limited sample size resulting from missing data in the paternal questionnaires. Therefore, strong interpretations of this finding are unwarranted without replication in studies with larger and more complete datasets.

Finally, we also explored whether particular parenting styles are associated with pacifier use. The self-report questionnaire we used to assess this dimension (Venuti and Senese, 2007), highlights three different types of parenting styles or ways in which the parent interacts with the child. The social type of interaction fosters the development of children’s social competencies; the didactic style promotes the child’s cognitive and linguistic development; and the discipline style encourages the development of attitudes complying with conventions and rules, as well as respect for authority. Pacifier use in this developmental period is not associated with any of these parenting styles.

As far as the child’s linguistic abilities are concerned, we did not observe a significant relationship between the use of the pacifier and the child’s first vocabulary. Several indices of the CDI questionnaire (such as the age of lexical development and the lexical quotient) were in line with the normative data and the characteristic variability of this developmental age, and they were not correlated to the months of pacifier use. One concern of this study regards the vocabulary scores obtained from the CDI. More than half of our sample scored below the 10th percentile on the CDI, which was a surprising finding. We considered several factors that might contribute to these unexpectedly low scores. Firstly, our data were collected between 2016 and 2017 from three nurseries in central Italy (two in a suburb of Rome, one in Venafro, Isernia province). While we aimed for diverse representation, it’s possible that the linguistic development and typical vocabulary acquisition patterns within these specific Italian contexts, particularly in a suburban or rural settings, may differ from the population on which the 2007 CDI norms were established. Secondly, the age of the CDI norms themselves (published in 2007) might play a role. Language use and exposure can evolve over time due to societal, technological, and cultural shifts. Therefore, it’s conceivable that applying norms developed nearly a decade prior to our data collection might not perfectly reflect contemporary vocabulary development, especially in a different geographical and cultural context. These factors suggest that the direct comparison of our sample’s raw CDI scores to the published norms should be interpreted with caution. Future research in similar populations would benefit from the use of more recently updated and culturally specific normative data.

When examining unique word production, our model showed no significant distinction between the Higher (Group 2) and Lower (Group 1) Pacifier Use groups, after controlling for the influence of age. This suggests that pacifier use, as categorized in this study, may not be a primary factor differentiating unique word production rates between these groups. Importantly, and in line with typical language development, a significant increase in unique word production was evident with advancing age, independent of pacifier use.

While some literature, including the recent review by Kanellopoulos and Costello (2024), highlights associations between extensive pacifier use and smaller vocabulary sizes in early childhood, our study did not find a significant effect of pacifier use on children’s vocabulary. This discrepancy can be attributed to several factors inherent in the design and scope of our research, as well as the broader context of existing literature. Firstly, a key limitation of our study was the reliance on general parental self-report for pacifier use, which precluded the collection of context-specific data (e.g., frequency of daytime vs. nighttime use, duration during interactive vs. solitary play). Kanellopoulos and Costello (2024) emphasize that the timing and context of pacifier use, rather than mere presence, are crucial determinants of its influence on linguistic capabilities. Our global measure of pacifier use might not have been sensitive enough to capture these nuanced effects, particularly the potential interference with social-linguistic interactions during critical learning periods. Studies that quantify daytime usage hours, for instance, often find them to be more relevant to speech development than nighttime usage (Strutt et al., 2021). Without this granular data, any observed effect might be diluted or masked. Secondly, the mixed outcomes in the literature itself (as acknowledged by Kanellopoulos and Costello, 2024) underscore the complexity of this relationship. Methodological differences across studies, such as variations in sample characteristics (e.g., age range, socioeconomic background, developmental stage), measures of pacifier use, and assessment of vocabulary, can lead to divergent findings. Our specific sample characteristics and the vocabulary measure used might have influenced our ability to detect a significant effect. Furthermore, it’s possible that the age range of our participants or the specific linguistic aspect of vocabulary we assessed (e.g., overall vocabulary production/comprehension) was less susceptible to the effects of pacifier use than other language dimensions or developmental stages. Some studies might detect effects on specific phonetic or articulatory development that do not immediately translate to overall vocabulary size as measured by tools like the CDI, or effects might be more pronounced at different developmental windows. In conclusion, while our findings do not show a significant direct effect of general pacifier use on vocabulary, this should be interpreted within the context of our methodological approach, particularly the lack of detailed contextual information on pacifier use. This highlights the ongoing need for future research to employ more precise and context-sensitive measures of pacifier use to fully understand its potential impact on specific aspects of early language development.

Beyond overall vocabulary, we also explored potential influences on specific word categories investigating the relationship between pacifier use (Group 1: Lower Pacifier Use; Group 2: Higher Pacifier Use) and the number of unique words produced by children, while controlling for age. Age consistently emerged as a highly significant positive predictor for the acquisition of unique words in almost all examined lexical categories. This finding aligns with developmental expectations, confirming that children’s vocabulary generally expands with increasing age. For the vast majority of lexical categories, there was no statistically significant difference in the number of unique words produced between children who used a pacifier for longer (Group 2) and those who used it less (Group 1). This suggests that pacifier use, as defined by the ‘percentage of usage’ in this analysis, does not appear to have a widespread negative (or positive) impact on unique word acquisition across these categories. This also means that our hypothesis of a possible modulation of vocabulary composition based on pacifier use (i.e., a paucity of the first abstract words according to pacifier use) has not been confirmed by current data. The only category where pacifier use showed a statistically significant effect was Routines. In this category, children in Group 2 (longer pacifier users) demonstrated a significantly higher number of unique words related to routines compared to Group 1. This is an interesting finding that warrants further exploration to understand the underlying mechanisms or contextual factors that might explain this particular association. A few categories, such as Auxiliary (for Group 2), Familiar Objects, and Verbs (for age), showed marginally significant *p*-values (e.g., *p* ≈ 0.05–0.06), suggesting a potential trend that could be further investigated with larger sample sizes or different analytical approaches. For the Articles and Conjunctions categories, neither age nor pacifier use demonstrated a significant impact on the number of unique words, suggesting that the acquisition of these specific grammatical categories might follow different developmental trajectories or be less influenced by the demographic and behavioral factors examined in this study. Overall, our findings predominantly indicate that age is the strongest determinant of vocabulary growth across most lexical categories in this sample. While pacifier use does not generally appear to hinder unique word production, its specific association with a higher number of unique words in the routines category presents an intriguing area for future research.

To further assess the dimension of abstractness in the child’s early vocabulary (which is typically characterized by concrete words and less by abstract words), we obtained abstractness ratings for the majority of the words in CDI vocabulary list. Our analysis indicated that children in the group with higher pacifier use (equal or more than 76% of their lives) did not show a statistically significant difference in the mean abstractness of their produced vocabulary compared to those with lower pacifier use (less than 76% of their lives), once age was considered. Furthermore, age itself did not significantly influence the mean abstractness of produced vocabulary in our sample, suggesting that vocabulary abstractness did not change notably with increasing age within our study population (between 24 and 36 months of age).

The reason why we did not replicate our previous results on abstract concepts with younger children is multifold. First, in previous studies, we tested the long-term effects of pacifier use, and specifically, the effects of long-term pacifier use on the definition and processing of abstract concepts in children beyond age three. In contrast, in the current study, we directly test the effect of a pacifier while it is used. Second, it is possible that the effect is not present because most abstract concepts are acquired later, after age four, building on previously acquired words (Ponari et al., 2018). A recent microgenetic study on parent-infant interactions shows that, during the second year of life, children use the first abstract words. Still, they are very simple ones (e.g., routines for affirming and negating (“yes”, “no”), verbs referring to actions, and routines like “all done”: and “all gone”) (Bellagamba et al., 2022). While the social dimension might be crucial for the acquisition of these early concepts (see also Bergelson and Swingley, 2013), language likely does not play a major role, in contrast to abstract concepts acquired by school-age children, more similar to adults (e.g., “justice”) (Gleitman et al., 2005). Finally, some of the mechanisms that we posit as crucial for abstract concepts processing, as the inner search for meaning, the inner re-explanation of the word meaning, and the reliance on others to ask for information and support, likely occur through inner speech, which develops later than age three (Alderson-Day and Fernyhough, 2015).

More generally, we did not find an effect of pacifier use on language development overall. The evidence of a detrimental effect of pacifier use on speech and language impairment is inconsistent [see (Nelson, 2012) for a review] and related to its prolonged use (i.e., for more than three years of age). For example, in Barbosa et al. (2009) prolonged pacifier use was associated with speech disorders in preschool children. The negative effects of the pacifier tend to self-reduce after its withdrawal so that breathing and speech functions increase in 4 years children 12 months after withdrawal (Scudine et al., 2021). Nevertheless, some alterations in masticatory functions seem to persist even a year after abandoning the pacifier (Scudine et al., 2022), suggesting that three years of age (or earlier) might be a critical set point for pacifier withdrawal.

### 4.1 Limitations and future directions

Our study, while offering valuable insights, is subject to some methodological limitations. A primary concern involves the sample size and the presence of missing data. While our sample (*N* = 98) is comparable to previous studies using similar instruments (e.g., Cattani and Celik, 2024), a formal *a priori* power analysis was not conducted due to feasibility constraints. More significantly, a substantial proportion of missing data (e.g., 24.5% for maternal efficacy, 62–66% for paternal measures) led to smaller effective subsamples for some analyses. Although we employed Bayesian statistics, which directly quantify evidence, the generalizability and robustness of some findings, particularly regarding paternal sense of efficacy, should be interpreted with caution. This pattern of missingness for parental characteristics might reflect a reluctance to provide personal information about their parenting role and attitudes, potentially exacerbated by the requirement to return materials to the child’s school.

Furthermore, our reliance on parental self-report introduces several potential biases. For instance, social desirability bias may have led parents to underreport the frequency or duration of pacifier use, potentially attenuating any observed negative associations with language development. Similarly, reporting bias regarding children’s vocabulary size on the CDI is a concern; parents’ perceptions can be influenced by expectations or comparisons, leading to over-or underestimation of their child’s actual vocabulary. Recall bias is also relevant, as parents might not accurately remember past pacifier use or precise vocabulary acquisition ages, potentially obscuring true relationships. Consequently, observed associations should be interpreted with caution, acknowledging the inherent limitations of self-report data.

Another important point is the scope of our language assessment. We focused solely on early vocabulary characteristics (e.g., number of words produced, categories) and did not include measures of speech production. Previous research, while sometimes inconsistent, suggests a detrimental effect of pacifier use on speech production in preschool children [e.g., (Barbosa et al., 2009; Strutt et al., 2021)]. Given the pacifier’s impact on articulatory motility, it’s plausible that an effect might emerge on speech production, a dimension not fully explored in our 18–36 month age range. Similarly, our reliance on general self-reported pacifier use prevents definitive conclusions about its specific impact on social-linguistic interactions in various contexts (e.g., awake vs. sleep), with daytime usage likely being more relevant for speech development (Strutt et al., 2021).

Finally, our linear regression model on vocabulary abstractness ratings did not reveal a statistically significant association with pacifier use duration or age, nor did it explain substantial variance. This may, in part, be due to the MacArthur-Bates CDI’s inherent skew towards concrete vocabulary, which might have limited its sensitivity in comprehensively assessing abstract word acquisition and potentially obscured subtle relationships. Previous works point to a detrimental effect of pacifier use on speech production in preschool children, although with some inconsistencies (Baker et al., 2018; Burr et al., 2021; Shotts et al., 2008). Here we did not consider such a dimension but only the characteristics of the first vocabulary (e.g., the number of words produced by the child and the different categories, according to parental reports). It is possible that if we consider the child’s speech—given the limitation that the pacifier exerts on the motility of the speech articulators—an effect of the pacifier might emerge. So, it remains to be tested a concurrent effect of pacifier use on speech production in children aged 18–36 months.

To address these limitations, future research would benefit from larger, more complete datasets to enhance statistical power and generalizability. Incorporating more objective measures of language development, such as direct assessments by trained professionals, could provide more precise and less biased insights. Longitudinal study designs tracking both pacifier use and language development over time with multiple assessment points would offer a more nuanced understanding of potential relationships. Furthermore, exploring the use of multiple informants (e.g., teachers, other caregivers) could provide a more comprehensive perspective on children’s language abilities. For vocabulary abstractness, future analyses could explore more complex statistical models, including interactions with other crucial environmental and child-specific factors (e.g., caregiver linguistic input, overall cognitive development). Despite the inherent limitations of parental report measures, such as the CDI, their feasibility and ability to capture broad language development in naturalistic settings remain valuable. Our study provides crucial preliminary insights that can inform future research employing more direct and objective measures. Building on these findings, future research could also help address the cross-cultural generalizability of our results by comparing data from Italian children with that of children from other cultures, particularly where the duration of pacifier use might differ.

## 5 Conclusion

In this study, we found no evidence of a detrimental effect of concurrent pacifier use on the breath of children’s first vocabulary or its composition. The absence of a selective effect on abstract concepts might be due to the late age of acquisition of most abstract words, which typically occurs later than age four, when linguistic competence is consolidated, and vocabulary is richer. The impact of pacifier use on the linguistic dimension seems to be relegated to its prolonged use and to the age of pacifier withdrawal, particularly critical beyond three years of age.

## Data Availability

The raw data supporting the conclusions of this article will be made available by the authors, without undue reservation.

## Footnotes

^1^These statistics are based on 14 participants, as one participant was excluded since she did not provide her date of birth.
