# Supplementary material for: Impact of pacifier use and parenting characteristics on toddlers’ vocabulary development
Source: Front Psychol. 2025 Jul 23;16:1599801. doi: 10.3389/fpsyg.2025.1599801 (PMC12325219; doi:10.3389/fpsyg.2025.1599801)
Supplement: Supplementary file 1 [file Table_1.docx]

**Appendix**

**Table 1** presents the distribution of the analyzed sample based on gender and pacifier use. Children were divided according to the chronological age of the sample into 18 groups (of different numbers).

|  | **Female** | | | **Male** | | | **NA** |
| --- | --- | --- | --- | --- | --- | --- | --- |
| **Age** | **Never** | **Yes, no more** | **Yes, still using** | **Never** | **Yes, no more** | **Yes, still using** | **Never** |
| **18** | 0 | 0 | 2 | 2 | 0 | 0 | 0 |
| **19** | 0 | 0 | 1 | 0 | 0 | 4 | 1 |
| **20** | 2 | 0 | 1 | 0 | 0 | 2 | 0 |
| **21** | 0 | 1 | 1 | 1 | 0 | 1 | 0 |
| **23** | 3 | 0 | 2 | 0 | 0 | 4 | 0 |
| **24** | 1 | 1 | 3 | 1 | 0 | 3 | 0 |
| **25** | 0 | 0 | 2 | 0 | 0 | 1 | 0 |
| **26** | 0 | 2 | 1 | 1 | 1 | 1 | 0 |
| **27** | 0 | 1 | 0 | 0 | 1 | 0 | 0 |
| **28** | 0 | 0 | 2 | 2 | 1 | 1 | 0 |
| **30** | 1 | 1 | 3 | 2 | 0 | 1 | 0 |
| **31** | 1 | 0 | 0 | 0 | 1 | 0 | 0 |
| **32** | 2 | 1 | 1 | 1 | 2 | 3 | 0 |
| **33** | 2 | 0 | 0 | 0 | 2 | 1 | 0 |
| **34** | 1 | 1 | 0 | 2 | 0 | 3 | 0 |
| **35** | 0 | 1 | 0 | 1 | 1 | 0 | 0 |
| **36** | 2 | 0 | 0 | 2 | 2 | 0 | 0 |
| **38** | 1 | 0 | 0 | 0 | 2 | 0 | 0 |
| **Tot** | **16** | **9** | **19** | **15** | **13** | **25** | **1** |
| ***Tot %*** | ***37*** | ***20*** | ***43*** | ***28*** | ***25*** | ***47*** |  |

**Table 2. Chronological age and total words produced with respect to the normative sample of the Italian CDI**

| Age (in months) | **<C5** | **C5** | **C10** | **C25** | **C50** | **C75** | **C90** | **C95** | **C96** | **Total** |
| --- | --- | --- | --- | --- | --- | --- | --- | --- | --- | --- |
| 18 | 1 | 1 |  | 1 |  | 1 |  |  |  | 4 |
| 19 | 1 |  | 2 | 1 | 1 | 1 |  |  |  | 6 |
| 20 |  | 1 | 1 | 3 |  |  |  |  |  | 5 |
| 21 | 2 |  |  | 1 | 1 |  |  |  |  | 4 |
| 22 | 1 |  | 1 | 1 |  | 1 |  |  |  | 4 |
| 23 | 2 |  | 3 |  | 3 |  | 1 |  |  | 9 |
| 24 | 2 | 2 |  | 2 |  |  |  |  |  | 6 |
| 25 | 2 |  | 2 |  |  |  |  | 1 |  | 5 |
| 26 | 1 |  |  | 1 | 1 |  | 1 |  |  | 4 |
| 27 | 2 | 1 |  |  | 1 | 1 |  |  |  | 5 |
| 28 | 1 |  | 2 | 1 |  |  | 1 |  |  | 5 |
| 29 |  |  |  |  | 1 |  |  |  |  | 1 |
| 30 | 3 | 1 | 1 | 2 | 1 | 2 |  | 1 |  | 11 |
| 31 | 1 |  |  |  |  |  |  |  |  | 1 |
| 32 | 4 |  |  |  | 1 |  | 2 |  |  | 7 |
| 33 | 2 |  | 2 | 2 |  | 1 |  |  |  | 7 |
| 34 | 1 | 1 |  |  | 2 |  |  |  |  | 4 |
| 35 | 1 | 1 |  | 1 | 1 | 1 |  |  |  | 5 |
| 36 |  | 1 |  |  | 2 |  |  |  | 1 | 4 |
| 38 |  | 1 |  |  |  |  |  |  |  | 1 |
| **Total** | **27** | **10** | **14** | **16** | **15** | **8** | **5** | **2** | **1** | **98** |

**Abstractness Rating Task Instructions**

"In this study, we are selecting linguistic stimuli and need evaluations on a series of words to select them. The purpose of this experiment is to evaluate the degree of abstractness of a series of words. On the following pages, you will find a list of words. Each word is accompanied by a rating scale. The scale ranges from 1 to 7, as follows:

More Abstract 1 2 3 4 5 6 7 Less Abstract

Your task is to rate how abstract the word is. Please keep in mind that the value of '1' corresponds to more abstract and '7' to less abstract. Feel free to use the entire range of values provided. In expressing your ratings, try to be as precise as possible, but do not spend too much time on a single word."

**Table 3.** Abstractness Ratings for CDI Italian Form Vocabulary

Items as Judged by Adult Native Speakers.

| **Category of PVB** | **Stimulus** | **mean** | **standard deviation** |
| --- | --- | --- | --- |
| Adjectives and qualities | addormentato | 4,47 | 2,00 |
| Adjectives and qualities | alto | 4,47 | 1,46 |
| Adjectives and qualities | amaro | 4,67 | 1,95 |
| Adjectives and qualities | arancione | 5,00 | 1,60 |
| Adjectives and qualities | arrabbiato | 4,33 | 1,80 |
| Adjectives and qualities | asciutto | 5,40 | 1,30 |
| Adjectives and qualities | attento | 3,60 | 1,64 |
| Adjectives and qualities | bagnato | 5,40 | 1,12 |
| Adjectives and qualities | bello | 3,27 | 1,53 |
| Adjectives and qualities | bianco | 4,67 | 1,80 |
| Adjectives and qualities | blu | 4,67 | 1,80 |
| Adjectives and qualities | brutto | 3,20 | 1,70 |
| Adjectives and qualities | buio | 4,93 | 1,62 |
| Adjectives and qualities | buono | 3,50 | 2,03 |
| Adjectives and qualities | caldo | 4,60 | 1,64 |
| Adjectives and qualities | calmo/tranquillo | 3,40 | 1,76 |
| Adjectives and qualities | carino | 3,33 | 1,76 |
| Adjectives and qualities | cattivo | 3,20 | 1,74 |
| Adjectives and qualities | contento | 3,60 | 1,59 |
| Adjectives and qualities | corto | 4,27 | 1,67 |
| Adjectives and qualities | disubbidiente | 3,40 | 1,59 |
| Adjectives and qualities | dolce | 4,13 | 2,03 |
| Adjectives and qualities | duro | 4,73 | 1,49 |
| Adjectives and qualities | felice | 3,14 | 1,75 |
| Adjectives and qualities | ferito | 4,47 | 1,77 |
| Adjectives and qualities | finito | 3,40 | 1,76 |
| Adjectives and qualities | forte | 3,73 | 1,58 |
| Adjectives and qualities | freddo | 4,47 | 1,46 |
| Adjectives and qualities | gentile | 3,08 | 1,61 |
| Adjectives and qualities | giallo | 4,73 | 1,67 |
| Adjectives and qualities | grande/grosso | 4,20 | 1,47 |
| Adjectives and qualities | leggero | 4,07 | 1,53 |
| Adjectives and qualities | lento | 3,60 | 1,80 |
| Adjectives and qualities | lungo | 4,07 | 1,62 |
| Adjectives and qualities | malato | 3,67 | 1,72 |
| Adjectives and qualities | marrone | 4,53 | 1,77 |
| Adjectives and qualities | morbido | 4,53 | 1,51 |
| Adjectives and qualities | nero | 4,60 | 1,68 |
| Adjectives and qualities | nuovo | 3,27 | 1,49 |
| Adjectives and qualities | pazzo/matto | 2,80 | 1,37 |
| Adjectives and qualities | pesante | 4,27 | 1,44 |
| Adjectives and qualities | piano | 3,27 | 1,58 |
| Adjectives and qualities | piccolo | 4,00 | 1,60 |
| Adjectives and qualities | pieno | 4,13 | 1,51 |
| Adjectives and qualities | povero | 3,00 | 1,00 |
| Adjectives and qualities | pulito | 4,13 | 1,60 |
| Adjectives and qualities | rosso | 4,53 | 1,68 |
| Adjectives and qualities | rotto | 4,33 | 1,72 |
| Adjectives and qualities | salato | 4,73 | 1,75 |
| Adjectives and qualities | sbagliato | 3,00 | 1,60 |
| Adjectives and qualities | schifoso | 3,60 | 1,72 |
| Adjectives and qualities | sciocco/stupido | 3,13 | 1,41 |
| Adjectives and qualities | spaventato | 3,67 | 1,68 |
| Adjectives and qualities | sporco | 4,13 | 1,41 |
| Adjectives and qualities | stanco | 3,87 | 1,55 |
| Adjectives and qualities | stretto | 4,27 | 1,98 |
| Adjectives and qualities | sveglio | 4,27 | 1,67 |
| Adjectives and qualities | triste | 3,93 | 1,94 |
| Adjectives and qualities | ultimo | 3,27 | 1,91 |
| Adjectives and qualities | vecchio | 3,60 | 1,35 |
| Adjectives and qualities | veloce/svelto | 3,40 | 1,59 |
| Adjectives and qualities | verde | 4,53 | 1,68 |
| Adjectives and qualities | vuoto | 3,60 | 1,64 |
| Games and Routines | auguri | 2,53 | 1,25 |
| Games and Routines | basta | 2,40 | 1,40 |
| Games and Routines | bravo | 2,80 | 1,26 |
| Games and Routines | bua | 4,33 | 2,02 |
| Games and Routines | bum (cade) | 3,87 | 2,45 |
| Games and Routines | buonanotte/buongiorno | 3,00 | 2,00 |
| Games and Routines | che rumore/chiasso | 4,07 | 1,91 |
| Games and Routines | ciao | 3,20 | 2,08 |
| Games and Routines | cuccù-settete | 3,73 | 2,25 |
| Games and Routines | grazie/prego | 3,13 | 1,81 |
| Games and Routines | nanna | 4,33 | 2,02 |
| Games and Routines | no | 3,80 | 2,31 |
| Games and Routines | non c'è più/più | 3,00 | 1,60 |
| Games and Routines | per favore/per piacere | 2,87 | 1,81 |
| Games and Routines | pronto, chi è? (al telefono) | 3,73 | 2,22 |
| Games and Routines | un, due, tre | 3,07 | 1,39 |
| Games and Routines | via | 3,47 | 2,00 |
| Games and Routines | zitto/sch | 3,60 | 2,03 |
| Interrogatives | che/che cosa? | 3,47 | 2,07 |
| Interrogatives | chi? | 3,53 | 2,03 |
| Interrogatives | come? | 3,40 | 2,06 |
| Interrogatives | dove? | 3,80 | 2,11 |
| Interrogatives | perchè? | 3,20 | 2,14 |
| Interrogatives | quale? | 3,64 | 2,21 |
| Interrogatives | quando? | 3,33 | 2,13 |
| Sounds and voices of nature | bau bau | 4,73 | 2,05 |
| Sounds and voices of nature | beh beh | 4,40 | 2,06 |
| Sounds and voices of nature | brum brum | 5,00 | 1,96 |
| Sounds and voices of nature | cip cip | 4,73 | 2,05 |
| Sounds and voices of nature | clop clop | 3,93 | 2,05 |
| Sounds and voices of nature | coccodè | 4,87 | 2,00 |
| Sounds and voices of nature | grr | 3,53 | 2,03 |
| Sounds and voices of nature | ih oh | 4,60 | 2,03 |
| Sounds and voices of nature | miao | 4,93 | 1,79 |
| Sounds and voices of nature | muh | 4,27 | 2,12 |
| Sounds and voices of nature | qua qua | 4,73 | 2,05 |
| Sounds and voices of nature | tuttù | 2,93 | 2,02 |
| Adverbs Expressions of place and quantity | anche/pure | 2,33 | 1,11 |
| Adverbs Expressions of place and quantity | tutto | 2,93 | 1,39 |
| Adverbs Expressions of place and quantity | un pò | 2,60 | 1,24 |
| Adverbs Expressions of time | adesso/ora | 3,20 | 2,04 |
| Adverbs Expressions of time | domani | 3,13 | 1,96 |
| Adverbs Expressions of time | dopo/poi | 3,00 | 1,85 |
| Adverbs Expressions of time | giorno | 3,47 | 2,20 |
| Adverbs Expressions of time | ieri | 3,20 | 2,04 |
| Adverbs Expressions of time | mattina | 3,20 | 1,82 |
| Adverbs Expressions of time | notte | 3,40 | 1,84 |
| Adverbs Expressions of time | oggi | 3,47 | 2,13 |
| Adverbs Expressions of time | pomeriggio | 3,07 | 1,83 |
| Adverbs Expressions of time | presto | 2,27 | 0,96 |
| Adverbs Expressions of time | sera | 3,07 | 1,67 |
| Adverbs Expressions of time | tardi | 2,13 | 1,06 |
| Games and Routines | dare le tottò | 4,20 | 1,74 |
| Games and Routines | fare il bagno | 4,67 | 1,84 |
| Games and Routines | fare la pipì | 4,73 | 1,91 |
| Games and Routines | fare la popò | 4,73 | 1,91 |
| Games and Routines | giro-girotondo | 4,13 | 1,96 |
| Games and Routines | voler bene | 2,87 | 1,85 |
| Clothing | bavaglino | 6,71 | 0,83 |
| Clothing | borsa | 6,67 | 0,82 |
| Clothing | bottone | 6,73 | 0,80 |
| Clothing | bretelle | 6,73 | 0,80 |
| Clothing | calze | 6,73 | 0,80 |
| Clothing | calzettoni | 6,73 | 0,80 |
| Clothing | camicia | 6,67 | 0,82 |
| Clothing | cappello | 6,73 | 0,59 |
| Clothing | cappotto | 6,73 | 0,59 |
| Clothing | cinta | 6,73 | 0,80 |
| Clothing | collana | 6,73 | 0,59 |
| Clothing | giacca | 6,73 | 0,59 |
| Clothing | gonna | 6,73 | 0,59 |
| Clothing | grembiule | 6,73 | 0,59 |
| Clothing | guanti | 6,73 | 0,59 |
| Clothing | jeans | 6,73 | 0,80 |
| Clothing | maglione | 6,73 | 0,59 |
| Clothing | mutande | 6,73 | 0,59 |
| Clothing | occhiali | 6,80 | 0,41 |
| Clothing | pannolino | 6,73 | 0,80 |
| Clothing | pantaloni | 6,73 | 0,59 |
| Clothing | pantofole | 6,64 | 0,84 |
| Clothing | pigiama | 6,73 | 0,59 |
| Clothing | scarpe | 6,67 | 0,72 |
| Clothing | sciarpa | 6,67 | 0,82 |
| Clothing | stivali | 6,73 | 0,80 |
| Clothing | tuta | 6,71 | 0,83 |
| Clothing | vestito | 6,53 | 0,92 |
| Outdoors | albero | 6,80 | 0,56 |
| Outdoors | altalena | 6,87 | 0,35 |
| Outdoors | ascensore | 6,73 | 0,80 |
| Outdoors | bandiera | 6,53 | 1,06 |
| Outdoors | benzina | 5,87 | 1,55 |
| Outdoors | campana | 6,53 | 1,30 |
| Outdoors | cielo | 5,47 | 1,85 |
| Outdoors | erba | 6,60 | 0,74 |
| Outdoors | fiore | 6,73 | 0,70 |
| Outdoors | foglia | 6,60 | 0,83 |
| Outdoors | fontana | 6,60 | 0,74 |
| Outdoors | fumo | 5,60 | 1,40 |
| Outdoors | garage | 6,53 | 0,83 |
| Outdoors | luna | 5,47 | 1,88 |
| Outdoors | nebbia | 4,80 | 2,04 |
| Outdoors | neve | 6,27 | 1,03 |
| Outdoors | nuvola | 4,80 | 2,18 |
| Outdoors | pioggia | 6,13 | 0,99 |
| Outdoors | piscina | 6,73 | 0,59 |
| Outdoors | pompa | 6,53 | 1,06 |
| Outdoors | prato | 6,60 | 0,63 |
| Outdoors | sasso | 6,80 | 0,56 |
| Outdoors | scivolo | 6,87 | 0,52 |
| Outdoors | sole | 5,53 | 1,41 |
| Outdoors | stella | 5,20 | 1,82 |
| Outdoors | strada | 6,47 | 0,99 |
| Outdoors | terra | 6,27 | 1,10 |
| Outdoors | tetto | 6,53 | 0,74 |
| Outdoors | torre | 6,43 | 1,16 |
| Outdoors | tubo | 6,47 | 1,25 |
| Outdoors | vento | 4,93 | 1,75 |
| Animals | agnello | 6,93 | 0,26 |
| Animals | animale | 6,00 | 1,20 |
| Animals | ape | 6,93 | 0,26 |
| Animals | asino | 6,93 | 0,26 |
| Animals | cane | 6,93 | 0,26 |
| Animals | capra | 6,93 | 0,26 |
| Animals | cavallo | 6,93 | 0,27 |
| Animals | cerbiatto | 6,93 | 0,26 |
| Animals | coccodrillo | 6,93 | 0,26 |
| Animals | coniglio | 6,93 | 0,26 |
| Animals | cucciolo | 5,67 | 1,05 |
| Animals | elefante | 6,93 | 0,26 |
| Animals | farfalla | 6,87 | 0,35 |
| Animals | foca | 6,93 | 0,26 |
| Animals | formica | 6,93 | 0,26 |
| Animals | gallina | 6,93 | 0,26 |
| Animals | gallo | 6,93 | 0,26 |
| Animals | gatto | 6,93 | 0,26 |
| Animals | giraffa | 6,93 | 0,26 |
| Animals | gufo | 6,93 | 0,26 |
| Animals | ippopotamo | 6,93 | 0,27 |
| Animals | leone | 6,93 | 0,26 |
| Animals | lupo | 6,93 | 0,26 |
| Animals | maiale | 6,93 | 0,26 |
| Animals | mosca | 6,93 | 0,26 |
| Animals | mucca | 6,93 | 0,26 |
| Animals | oca | 6,93 | 0,26 |
| Animals | orso | 6,93 | 0,26 |
| Animals | papera | 6,93 | 0,27 |
| Animals | pecora | 6,93 | 0,26 |
| Animals | pesciolino | 6,40 | 0,91 |
| Animals | pinguino | 6,93 | 0,26 |
| Animals | pulcino | 6,93 | 0,26 |
| Animals | rana | 6,93 | 0,26 |
| Animals | scimmia | 6,93 | 0,26 |
| Animals | scoiattolo | 6,93 | 0,26 |
| Animals | tacchino | 6,87 | 0,35 |
| Animals | tartaruga | 6,93 | 0,26 |
| Animals | tigre | 6,93 | 0,26 |
| Animals | topo | 6,93 | 0,27 |
| Animals | uccellino | 6,47 | 0,92 |
| Animals | zanzara | 6,93 | 0,26 |
| Animals | zebra | 6,93 | 0,26 |
| Food and drinks | acqua | 6,80 | 0,41 |
| Food and drinks | arancia | 7,00 | 0,00 |
| Food and drinks | banana | 7,00 | 0,00 |
| Food and drinks | biscotti | 6,87 | 0,35 |
| Food and drinks | budino | 6,87 | 0,52 |
| Food and drinks | burro | 6,87 | 0,52 |
| Food and drinks | cacao | 6,67 | 0,72 |
| Food and drinks | caffè | 6,73 | 0,59 |
| Food and drinks | camomilla | 6,80 | 0,56 |
| Food and drinks | caramella | 6,87 | 0,35 |
| Food and drinks | carne | 6,60 | 0,74 |
| Food and drinks | carote | 6,87 | 0,35 |
| Food and drinks | ciliege | 6,87 | 0,35 |
| Food and drinks | cioccolata | 6,87 | 0,35 |
| Food and drinks | coca-cola | 6,67 | 0,62 |
| Food and drinks | cocomero/anguria | 7,00 | 0,00 |
| Food and drinks | cracker | 6,87 | 0,35 |
| Food and drinks | crema | 6,60 | 0,63 |
| Food and drinks | fagioli | 6,87 | 0,35 |
| Food and drinks | fagiolini | 6,93 | 0,26 |
| Food and drinks | formaggio | 6,80 | 0,56 |
| Food and drinks | fragola | 7,00 | 0,00 |
| Food and drinks | gelato | 6,87 | 0,35 |
| Food and drinks | ghiaccio | 6,67 | 0,62 |
| Food and drinks | gomma da masticare | 6,40 | 1,30 |
| Food and drinks | kiwi | 6,93 | 0,26 |
| Food and drinks | latte | 6,73 | 0,59 |
| Food and drinks | leccalecca | 6,93 | 0,26 |
| Food and drinks | mandarino | 6,93 | 0,26 |
| Food and drinks | marmellata | 6,80 | 0,41 |
| Food and drinks | mela | 6,93 | 0,26 |
| Food and drinks | melone | 7,00 | 0,00 |
| Food and drinks | miele | 6,73 | 0,59 |
| Food and drinks | minestrone/brodo | 6,67 | 0,49 |
| Food and drinks | noccioline | 6,87 | 0,35 |
| Food and drinks | olio | 6,67 | 0,62 |
| Food and drinks | pane | 6,87 | 0,35 |
| Food and drinks | panino | 6,87 | 0,35 |
| Food and drinks | panna | 6,73 | 0,46 |
| Food and drinks | pappa | 5,87 | 1,41 |
| Food and drinks | pasta | 6,80 | 0,41 |
| Food and drinks | patate | 6,87 | 0,35 |
| Food and drinks | patatine | 6,67 | 0,82 |
| Food and drinks | pera | 7,00 | 0,00 |
| Food and drinks | pesca | 6,93 | 0,26 |
| Food and drinks | piselli | 6,87 | 0,35 |
| Food and drinks | pizza | 6,80 | 0,41 |
| Food and drinks | pollo | 6,87 | 0,35 |
| Food and drinks | polpette | 6,87 | 0,52 |
| Food and drinks | pomodori | 6,93 | 0,26 |
| Food and drinks | riso | 6,87 | 0,35 |
| Food and drinks | sale | 6,73 | 0,59 |
| Food and drinks | spaghetti | 6,93 | 0,26 |
| Food and drinks | spinaci | 6,87 | 0,35 |
| Food and drinks | succo di frutta | 6,60 | 0,51 |
| Food and drinks | sugo | 6,87 | 0,35 |
| Food and drinks | the | 6,67 | 0,62 |
| Food and drinks | tonno | 6,80 | 0,56 |
| Food and drinks | torta | 6,79 | 0,43 |
| Food and drinks | tortellini | 6,87 | 0,52 |
| Food and drinks | uovo | 6,87 | 0,35 |
| Food and drinks | uva | 7,00 | 0,00 |
| Food and drinks | verdura | 6,33 | 0,98 |
| Food and drinks | vino | 6,20 | 1,42 |
| Food and drinks | yogurt | 6,73 | 0,59 |
| Food and drinks | zucchero | 6,67 | 0,72 |
| Food and drinks | zucchine | 6,93 | 0,27 |
| Toys | bambola | 6,53 | 0,83 |
| Toys | birilli | 6,73 | 0,59 |
| Toys | casetta | 5,93 | 1,49 |
| Toys | costruzioni | 6,00 | 1,51 |
| Toys | cubi | 6,47 | 1,13 |
| Toys | favola/storia | 3,47 | 1,81 |
| Toys | giocattolo | 5,93 | 1,39 |
| Toys | paletta | 6,80 | 0,41 |
| Toys | palla | 6,87 | 0,35 |
| Toys | palloncino | 6,79 | 0,43 |
| Toys | pistola | 6,67 | 0,72 |
| Toys | pongo/didò | 6,33 | 1,35 |
| Toys | pupazzi | 6,07 | 1,22 |
| Toys | secchiello | 6,80 | 0,56 |
| Toys | soldatini | 6,33 | 1,40 |
| Toys | tamburo | 6,80 | 0,41 |
| Toys | tromba | 6,67 | 0,72 |
| Toys | trottola | 6,73 | 0,70 |
| Food and drinks | aranciata | 6,47 | 1,13 |
| Furniture, rooms, and objects of the house | armadio | 6,73 | 0,80 |
| Furniture, rooms, and objects of the house | bagno | 6,60 | 0,63 |
| Furniture, rooms, and objects of the house | bidè | 6,80 | 0,77 |
| Furniture, rooms, and objects of the house | camera | 6,20 | 0,77 |
| Furniture, rooms, and objects of the house | cantina | 6,27 | 0,96 |
| Furniture, rooms, and objects of the house | cassetto | 6,67 | 0,72 |
| Furniture, rooms, and objects of the house | cucina | 6,33 | 0,90 |
| Furniture, rooms, and objects of the house | culla | 6,87 | 0,52 |
| Furniture, rooms, and objects of the house | divano | 6,87 | 0,52 |
| Furniture, rooms, and objects of the house | doccia | 6,60 | 0,74 |
| Furniture, rooms, and objects of the house | ferro da stiro | 6,53 | 1,25 |
| Furniture, rooms, and objects of the house | finestra | 6,40 | 1,35 |
| Furniture, rooms, and objects of the house | fon | 6,67 | 0,90 |
| Furniture, rooms, and objects of the house | forno | 6,71 | 0,83 |
| Furniture, rooms, and objects of the house | frigorifero | 6,73 | 0,80 |
| Furniture, rooms, and objects of the house | lavandino | 6,80 | 0,77 |
| Furniture, rooms, and objects of the house | lavatrice | 6,67 | 0,90 |
| Furniture, rooms, and objects of the house | letto | 6,80 | 0,56 |
| Furniture, rooms, and objects of the house | muro | 6,67 | 0,62 |
| Furniture, rooms, and objects of the house | poltrona | 6,87 | 0,52 |
| Furniture, rooms, and objects of the house | porta | 6,87 | 0,52 |
| Furniture, rooms, and objects of the house | quadro | 6,27 | 1,28 |
| Furniture, rooms, and objects of the house | radio | 6,40 | 1,12 |
| Furniture, rooms, and objects of the house | registratore | 6,33 | 1,11 |
| Furniture, rooms, and objects of the house | salotto | 6,53 | 0,64 |
| Furniture, rooms, and objects of the house | scala | 6,67 | 0,49 |
| Furniture, rooms, and objects of the house | sedia | 6,80 | 0,56 |
| Furniture, rooms, and objects of the house | seggiolone | 6,87 | 0,52 |
| Furniture, rooms, and objects of the house | specchio | 6,80 | 0,41 |
| Furniture, rooms, and objects of the house | tavolo | 6,80 | 0,56 |
| Furniture, rooms, and objects of the house | televisione/TV | 6,60 | 1,06 |
| Furniture, rooms, and objects of the house | terrazza | 6,47 | 0,83 |
| Furniture, rooms, and objects of the house | vasca da bagno | 6,87 | 0,52 |
| Household objects | asciugamano | 6,93 | 0,26 |
| Household objects | aspirapolvere | 6,60 | 0,91 |
| Household objects | biberon | 6,80 | 0,56 |
| Household objects | bicchiere | 6,87 | 0,35 |
| Household objects | bottiglia | 6,87 | 0,35 |
| Household objects | candeline | 6,80 | 0,56 |
| Household objects | cestino | 6,80 | 0,56 |
| Household objects | chiave | 6,80 | 0,56 |
| Household objects | ciuccio | 6,80 | 0,41 |
| Household objects | colori | 5,67 | 1,45 |
| Household objects | coltello | 6,53 | 1,36 |
| Household objects | coperchio | 6,80 | 0,56 |
| Household objects | coperta | 6,80 | 0,41 |
| Household objects | cucchiaio | 6,93 | 0,26 |
| Household objects | cuscino | 6,93 | 0,26 |
| Household objects | dentifricio | 6,80 | 0,77 |
| Household objects | disegno | 5,87 | 1,55 |
| Household objects | fazzoletto | 6,80 | 0,41 |
| Household objects | forbici | 6,53 | 1,25 |
| Household objects | forchetta | 6,60 | 1,12 |
| Household objects | fotografia | 6,20 | 1,15 |
| Household objects | giornale | 6,60 | 0,91 |
| Household objects | libro | 6,80 | 0,56 |
| Household objects | luce | 5,80 | 1,21 |
| Household objects | martello | 6,80 | 0,77 |
| Household objects | matita/penna | 6,87 | 0,35 |
| Household objects | medicina | 5,53 | 1,60 |
| Household objects | musica | 5,00 | 1,93 |
| Household objects | ombrello | 6,87 | 0,52 |
| Household objects | orologio | 6,47 | 1,13 |
| Household objects | pentola | 6,80 | 0,56 |
| Household objects | pettine | 6,93 | 0,26 |
| Household objects | pianta | 6,67 | 0,82 |
| Household objects | piatto | 6,80 | 0,56 |
| Household objects | sacchetto | 6,67 | 0,62 |
| Household objects | sapone | 6,60 | 0,74 |
| Household objects | scatola | 6,80 | 0,56 |
| Household objects | scopa | 6,80 | 0,56 |
| Household objects | secchio | 6,80 | 0,56 |
| Household objects | shampoo | 6,67 | 0,62 |
| Household objects | soldi | 5,43 | 1,55 |
| Household objects | spazzolino da denti | 6,93 | 0,26 |
| Household objects | straccio | 6,73 | 0,59 |
| Household objects | tappeto | 6,80 | 0,56 |
| Household objects | tappo | 6,80 | 0,41 |
| Household objects | tazza | 6,87 | 0,52 |
| Household objects | telefono | 6,80 | 0,41 |
| Household objects | termometro | 6,53 | 1,13 |
| Household objects | termosifone | 6,80 | 0,56 |
| Body parts | bocca | 6,93 | 0,26 |
| Body parts | braccio | 6,93 | 0,26 |
| Body parts | capelli | 6,87 | 0,35 |
| Body parts | caviglia | 6,93 | 0,26 |
| Body parts | denti | 7,00 | 0,00 |
| Body parts | dito | 7,00 | 0,00 |
| Body parts | faccia | 6,87 | 0,35 |
| Body parts | gambe | 6,93 | 0,26 |
| Body parts | ginocchio | 7,00 | 0,00 |
| Body parts | gola | 6,80 | 0,41 |
| Body parts | guance | 7,00 | 0,00 |
| Body parts | labbra | 7,00 | 0,00 |
| Body parts | lingua | 6,87 | 0,35 |
| Body parts | mano | 7,00 | 0,00 |
| Body parts | naso | 7,00 | 0,00 |
| Body parts | occhio | 6,93 | 0,26 |
| Body parts | ombelico | 6,87 | 0,35 |
| Body parts | orecchio | 7,00 | 0,00 |
| Body parts | organo genitale femminile | 6,73 | 0,59 |
| Body parts | organo genitale maschile | 6,73 | 0,59 |
| Body parts | pancia | 6,87 | 0,35 |
| Body parts | piede | 6,93 | 0,26 |
| Body parts | sederino | 6,53 | 0,92 |
| Body parts | seno | 6,87 | 0,52 |
| Body parts | spalla | 6,87 | 0,35 |
| Body parts | testa | 6,93 | 0,26 |
| Body parts | unghie | 6,87 | 0,35 |
| People | "il suo nome" | 4,60 | 1,76 |
| People | amico/a | 4,60 | 2,13 |
| People | babysitter/tata | 5,13 | 1,88 |
| People | bambino/a | 5,33 | 1,95 |
| People | barbiere | 5,47 | 1,64 |
| People | cugino/a | 4,87 | 1,85 |
| People | donna | 5,07 | 2,05 |
| People | dottore | 5,40 | 1,64 |
| People | fratello | 5,27 | 1,53 |
| People | maestro/a | 5,00 | 1,73 |
| People | mamma | 6,00 | 1,46 |
| People | nonna | 5,67 | 1,45 |
| People | nonno | 5,60 | 1,45 |
| People | panettiere | 5,40 | 1,45 |
| People | papà | 6,00 | 1,46 |
| People | parrucchiere | 5,47 | 1,46 |
| People | pediatra | 5,33 | 1,59 |
| People | persone | 4,60 | 2,13 |
| People | poliziotto | 5,40 | 1,45 |
| People | portiere | 5,40 | 1,45 |
| People | ragazzo/a | 4,80 | 1,90 |
| People | signore/a | 4,71 | 1,86 |
| People | soldato | 5,47 | 1,64 |
| People | sorella | 5,67 | 1,40 |
| People | uomo | 4,87 | 1,92 |
| People | vigile | 5,40 | 1,45 |
| People | zia | 5,33 | 1,54 |
| People | zio | 5,33 | 1,54 |
| Places to go | asilo | 6,50 | 0,76 |
| Places to go | bar | 6,47 | 0,92 |
| Places to go | bosco | 6,47 | 0,83 |
| Places to go | campagna | 6,07 | 0,88 |
| Places to go | casa | 6,67 | 0,72 |
| Places to go | chiesa | 6,40 | 0,99 |
| Places to go | circo | 6,33 | 0,98 |
| Places to go | città | 5,60 | 1,24 |
| Places to go | festa | 4,87 | 1,30 |
| Places to go | giardino | 6,27 | 0,88 |
| Places to go | giostra | 6,53 | 0,64 |
| Places to go | lavoro | 4,33 | 1,80 |
| Places to go | mare | 6,07 | 1,10 |
| Places to go | mercato | 6,13 | 0,92 |
| Places to go | montagna | 6,27 | 0,96 |
| Places to go | negozio | 6,27 | 0,88 |
| Places to go | ospedale | 6,27 | 0,96 |
| Places to go | parco giochi | 6,33 | 0,82 |
| Places to go | scuola | 6,20 | 1,15 |
| Places to go | spiaggia | 6,36 | 1,01 |
| Places to go | supermercato | 6,40 | 0,99 |
| Places to go | zoo | 6,40 | 0,83 |
| Vehicles | aereo | 6,80 | 0,41 |
| Vehicles | autobus | 6,87 | 0,35 |
| Vehicles | automobile | 6,80 | 0,41 |
| Vehicles | barca | 6,93 | 0,26 |
| Vehicles | bicicletta | 6,93 | 0,26 |
| Vehicles | camion | 6,87 | 0,35 |
| Vehicles | elicottero | 6,87 | 0,35 |
| Vehicles | gru | 6,73 | 0,59 |
| Vehicles | jeep | 6,80 | 0,56 |
| Vehicles | motocicletta | 6,87 | 0,35 |
| Vehicles | passeggino | 6,87 | 0,35 |
| Vehicles | slitta | 6,73 | 0,59 |
| Vehicles | trattore | 6,93 | 0,26 |
| Vehicles | treno | 6,87 | 0,35 |
| Prepositions | a | 2,47 | 1,25 |
| Prepositions | da | 2,53 | 1,30 |
| Prepositions | di | 2,47 | 1,25 |
| Prepositions | fra/tra | 2,93 | 1,49 |
| Prepositions and locatives | con | 2,73 | 1,49 |
| Prepositions and locatives | davanti | 3,60 | 1,96 |
| Prepositions and locatives | dentro | 3,60 | 1,76 |
| Prepositions and locatives | dietro | 3,60 | 1,96 |
| Prepositions and locatives | ecco | 2,40 | 1,72 |
| Prepositions and locatives | fuori | 3,53 | 1,68 |
| Prepositions and locatives | giù | 2,93 | 1,59 |
| Prepositions and locatives | in | 2,73 | 1,49 |
| Prepositions and locatives | li/là | 3,20 | 1,86 |
| Prepositions and locatives | lontano | 3,47 | 1,85 |
| Prepositions and locatives | per | 2,47 | 1,25 |
| Prepositions and locatives | qui/qua | 3,33 | 1,80 |
| Prepositions and locatives | sopra | 3,47 | 1,92 |
| Prepositions and locatives | sotto | 3,47 | 1,92 |
| Prepositions and locatives | su | 3,07 | 1,67 |
| Prepositions and locatives | vicino | 3,47 | 1,77 |
| Pronouns | che | 2,73 | 1,16 |
| Pronouns | ci | 3,67 | 1,84 |
| Pronouns | gli | 3,13 | 1,55 |
| Pronouns | io | 4,87 | 2,07 |
| Pronouns | lei | 4,27 | 1,94 |
| Pronouns | li/e | 3,07 | 1,62 |
| Pronouns | lo/a | 3,07 | 1,62 |
| Pronouns | loro | 4,27 | 1,94 |
| Pronouns | lui | 4,27 | 1,94 |
| Pronouns | me/mi | 4,21 | 1,81 |
| Pronouns | mio/a | 4,13 | 2,26 |
| Pronouns | noi | 4,60 | 1,99 |
| Pronouns | nostro/a | 4,00 | 2,14 |
| Pronouns | quello/a | 4,47 | 1,55 |
| Pronouns | questo/a | 4,53 | 1,60 |
| Pronouns | si | 3,87 | 2,03 |
| Pronouns | suo/a | 3,80 | 2,08 |
| Pronouns | te/ti | 3,73 | 1,87 |
| Pronouns | tu | 4,60 | 1,99 |
| Pronouns | tuo/a | 4,00 | 2,14 |
| Pronouns | vi | 3,53 | 1,68 |
| Pronouns | voi | 4,40 | 1,88 |
| Pronouns | vostro/a | 3,73 | 2,02 |
| Quantifiers | ancora | 2,53 | 1,46 |
| Quantifiers | di più | 3,07 | 1,44 |
| Quantifiers | molto | 2,47 | 1,19 |
| Quantifiers | nessuno | 2,93 | 1,58 |
| Quantifiers | niente | 2,73 | 1,67 |
| Quantifiers | poco | 2,93 | 1,62 |
| Quantifiers | tanto | 3,07 | 1,53 |
| Quantifiers | troppo | 2,80 | 1,37 |
| Games and Routines | telefonare | 5,27 | 1,71 |
| Verbs | abbracciare | 5,29 | 1,68 |
| Verbs | accendere | 4,93 | 1,44 |
| Verbs | acchiappare | 5,00 | 1,51 |
| Verbs | aggiustare | 5,00 | 1,46 |
| Verbs | aiutare | 4,07 | 1,91 |
| Verbs | alzarsi | 5,00 | 1,60 |
| Verbs | andare | 4,67 | 1,63 |
| Verbs | aprire | 5,07 | 1,53 |
| Verbs | arrampicarsi | 5,20 | 1,37 |
| Verbs | asciugare | 5,13 | 1,46 |
| Verbs | aspettare | 3,87 | 1,96 |
| Verbs | aver fame | 4,80 | 1,74 |
| Verbs | aver sete | 4,71 | 1,77 |
| Verbs | aver sonno | 4,40 | 1,92 |
| Verbs | baciare | 5,43 | 1,34 |
| Verbs | ballare | 5,27 | 1,33 |
| Verbs | bere | 5,73 | 1,39 |
| Verbs | bussare | 5,53 | 1,41 |
| Verbs | buttare | 5,27 | 1,53 |
| Verbs | cadere | 5,53 | 1,51 |
| Verbs | cercare | 4,47 | 1,64 |
| Verbs | chiudere | 5,27 | 1,67 |
| Verbs | colorare | 5,40 | 1,64 |
| Verbs | comprare | 5,00 | 1,51 |
| Verbs | conoscere | 3,60 | 1,96 |
| Verbs | coprire | 5,33 | 1,40 |
| Verbs | correre | 5,60 | 1,50 |
| Verbs | costruire | 5,60 | 1,40 |
| Verbs | cucinare | 5,36 | 1,28 |
| Verbs | cullare | 5,27 | 1,39 |
| Verbs | dare | 4,73 | 1,44 |
| Verbs | dare un calcio | 5,67 | 1,29 |
| Verbs | dire | 4,80 | 1,52 |
| Verbs | disegnare | 5,47 | 1,46 |
| Verbs | dondolare | 5,20 | 1,61 |
| Verbs | dormire | 5,40 | 1,80 |
| Verbs | entrare | 5,27 | 1,62 |
| Verbs | fare | 4,73 | 1,53 |
| Verbs | fermarsi | 5,07 | 1,75 |
| Verbs | finire | 4,27 | 1,44 |
| Verbs | giocare | 5,07 | 1,44 |
| Verbs | girare | 5,07 | 1,62 |
| Verbs | gridare/urlare | 5,47 | 1,30 |
| Verbs | guardare | 5,33 | 1,59 |
| Verbs | guidare | 5,14 | 1,75 |
| Verbs | lanciare | 5,33 | 1,40 |
| Verbs | lavare | 5,53 | 1,51 |
| Verbs | lavorare | 4,47 | 1,92 |
| Verbs | leccare | 5,67 | 1,35 |
| Verbs | leggere | 5,07 | 1,79 |
| Verbs | levare/togliere | 5,07 | 1,58 |
| Verbs | litigare | 4,80 | 1,42 |
| Verbs | mangiare | 5,80 | 1,37 |
| Verbs | mettere | 4,87 | 1,41 |
| Verbs | mordere | 5,53 | 1,51 |
| Verbs | nascondere/si | 4,80 | 1,78 |
| Verbs | nuotare | 5,60 | 1,55 |
| Verbs | parlare | 5,07 | 1,75 |
| Verbs | passeggiare | 5,20 | 1,57 |
| Verbs | pettinare | 5,43 | 1,50 |
| Verbs | piacere | 3,47 | 1,73 |
| Verbs | piangere | 5,33 | 1,68 |
| Verbs | portare | 4,87 | 1,68 |
| Verbs | prendere | 5,13 | 1,73 |
| Verbs | provare | 3,53 | 1,85 |
| Verbs | pulire | 5,47 | 1,46 |
| Verbs | raccontare | 4,20 | 1,86 |
| Verbs | regalare | 4,60 | 1,88 |
| Verbs | restare | 3,93 | 1,71 |
| Verbs | ridere | 5,47 | 1,55 |
| Verbs | rispondere | 5,07 | 1,62 |
| Verbs | rompere | 5,53 | 1,36 |
| Verbs | rovesciare | 5,53 | 1,36 |
| Verbs | saltare | 5,43 | 1,60 |
| Verbs | salutare | 5,00 | 1,56 |
| Verbs | sbrigarsi | 3,60 | 1,88 |
| Verbs | scappare | 4,93 | 1,62 |
| Verbs | scendere | 5,13 | 1,73 |
| Verbs | scrivere | 5,27 | 1,49 |
| Verbs | sedersi | 5,40 | 1,55 |
| Verbs | sentire | 4,33 | 2,09 |
| Verbs | soffiare | 5,33 | 1,45 |
| Verbs | spazzare | 5,33 | 1,59 |
| Verbs | spegnere | 5,20 | 1,70 |
| Verbs | spingere | 5,33 | 1,59 |
| Verbs | sporcarsi | 5,27 | 1,53 |
| Verbs | sputare | 5,43 | 1,50 |
| Verbs | stare | 4,07 | 1,83 |
| Verbs | strappare | 5,40 | 1,50 |
| Verbs | svegliarsi | 5,07 | 1,83 |
| Verbs | tagliare | 5,47 | 1,55 |
| Verbs | tenere | 4,67 | 1,72 |
| Verbs | tirare | 5,20 | 1,52 |
| Verbs | toccare | 5,87 | 1,25 |
| Verbs | trovare | 4,87 | 1,46 |
| Verbs | uscire | 5,07 | 1,67 |
| Verbs | vasino | 6,13 | 1,51 |
| Verbs | vedere | 5,47 | 1,46 |
| Verbs | venire | 4,93 | 1,49 |
| Verbs | versare | 5,33 | 1,59 |
| Verbs | volare | 4,27 | 2,12 |

**Table 4.** Estimates and Significance of Linguistic Categories Across Groups and Age

| **CATEGORY** | Group2 | | | | Age | | | |
| --- | --- | --- | --- | --- | --- | --- | --- | --- |
|  | Estimate | Std. Err | z value | Pr(>\|z\|) | Estimate | Std. Err | z value | Pr(>\|z\|) |
| Adjectivesqual_cat15 | 0.10895 | 0.19579 | 0.556 | 0.5779 | 0.09075 | 0.02505 | 3.623 | 0.000291 *** |
| Adverbs_cat16 | 0.06098 | 0.18126 | 0.336 | 0.73653 | 0.06539 | 0.02482 | 2.634 | 0.00843 ** |
| Advlocatquant_cat23 | -0.0535 | 0.17596 | -0.304 | 0.76123 | 0.08272 | 0.02392 | 3.458 | 0.000544 *** |
| Animals_cat2 | 0.08932 | 0.21008 | 1.02 | 0.30778 | 0.08932 | 0.02765 | 3.23 | 0.00124 ** |
| Ausiliars_cat21 | 0.4257 | 0.2278 | 1.869 | 0.06165 | 0.1047 | 0.0292 | 3.585 | 0.000338 *** |
| Bodyparts_cat7 | 0.0384 | 0.13925 | 0.276 | 0.78275 | 0.0413 | 0.01822 | 2.266 | 0.02344 * |
| Clothing_cat6 | -0.0729 | 0.17754 | -0.411 | 0.68127 | 0.06616 | 0.02313 | 2.861 | 0.00423 ** |
| Familiarobjects_cat8 | -0.0339 | 0.23682 | -0.143 | 0.886 | 0.05924 | 0.03099 | 1.911 | 0.056 |
| Fooddrinks_cat5 | 0.08225 | 0.19468 | 0.422 | 0.67268 | 0.07875 | 0.02533 | 3.109 | 0.00188 ** |
| Interrogatives_cat18 | -0.0738 | 0.15181 | -0.486 | 0.62671 | 0.06083 | 0.01955 | 3.112 | 0.00186 ** |
| Locations_cat11 | -0.0745 | 0.18503 | -0.402 | 0.6874 | 0.0779 | 0.02338 | 3.332 | 0.000863 *** |
| Outdoor_cat10 | -0.0408 | 0.18672 | -0.219 | 0.827 | 0.05361 | 0.02443 | 2.195 | 0.0282 * |
| People_cat12 | 0.14733 | 0.1444 | 1.02 | 0.3076 | 0.05606 | 0.01847 | 3.035 | 0.0024 ** |
| Pronouns_cat17 | 0.12422 | 0.18875 | 0.658 | 0.51047 | 0.07447 | 0.02491 | 2.99 | 0.00279 ** |
| Routines_cat13 | 0.24048 | 0.1072 | 2.243 | 0.02488 * | 0.04799 | 0.01361 | 3.527 | 0.00042 *** |
| Soundsvoices_cat1 | 0.11969 | 0.09934 | 1.205 | 0.22826 | 0.04182 | 0.01287 | 3.25 | 0.00116 ** |
| Toys_cat4 | 0.0098 | 0.17387 | 0.056 | 0.95505 | 0.0731 | 0.02269 | 3.222 | 0.00128 ** |
| Vehicles_cat3 | 0.13853 | 0.15994 | 0.866 | 0.38644 | 0.07869 | 0.02078 | 3.787 | 0.000152 *** |
| Verbs_cat14 | 0.15701 | 0.26824 | 0.585 | 0.5583 | 0.06868 | 0.03603 | 1.906 | 0.0566 |
| Furniture_cat9 | 0.03207 | 0.20313 | 0.158 | 0.8745 | 0.05386 | 0.02585 | 2.083 | 0.0372 * |
| Prepositions_cat19 | 0.08381 | 0.20151 | 0.416 | 0.67749 | 0.07575 | 0.02618 | 2.893 | 0.00381 ** |
| Articles_cat20 | -0.011 | 0.18412 | -0.06 | 0.9524 | -0.0004 | 0.02623 | -0.017 | 0.9865 |
| Conjunction_cat22 | -0.0287 | 0.17573 | -0.163 | 0.87 | 0.02105 | 0.02442 | 0.862 | 0.389 |

**Table Legend.** This table presents a statistical analysis of the linguistic categories derived from the CDI (Communicative Development Inventory). Each category, such as Adjectivesqual_cat15 (Qualitative Adjectives, CDI Category 15), Adverbs_cat16 (Adverbs, CDI Category 16), and so on, represents a distinct word type or semantic group as defined within the CDI framework.

For each linguistic category and variable combination, the table provides several statistical measures. The Estimate is the estimated coefficient from the statistical model, indicating the strength and direction of the relationship. The Std. Error represents the standard error of this estimate. The z value is the z-statistic, used to test the statistical significance of the estimate. Finally, Pr(>|z|) is the p-value, which indicates the probability of observing such a result if there were no true effect. A smaller p-value suggests stronger evidence against the null hypothesis, with common significance levels denoted by asterisks: *** for highly significant (p < 0.001), ** for very significant (p < 0.01), and * for significant (p < 0.05).
